# Caprine Kobuvirus VP1 Protein Activates the Mitochondrial Apoptotic Pathway via Interaction with BAD

**DOI:** 10.3390/vetsci13090993

**Published:** 2026-09-19

**Authors:** Kehamo Abi, Zhizhong Jing, Cheng Tang, Yao Wang, Kegu Ji’e, Yang Su, Falong Yang

**Affiliations:** 1 Key Laboratory of Veterinary Medicine of Universities in Sichuan, Southwest Minzu University, Chengdu 610041, China; abikehamo@swun.edu.cn (K.A.);; 2 State Key Laboratory of Veterinary Etiological Biology, Key Laboratory of Veterinary Public Health of Agriculture Ministry, Lanzhou Veterinary Research Institute, Chinese Academy of Agricultural Sciences, Lanzhou 730046, China

**Keywords:** caprine kobuvirus, structural protein VP1, pro-apoptotic protein BAD, mitochondrial pathway

## Abstract

Caprine kobuvirus (CKoV) is a member of the *Picornaviridae* family that causes enteritis in goats and poses a significant health threat, yet its molecular pathogenesis remains largely unknown. Here, we show that experimental infection of goats with CKoV induces apoptosis in the spleen, lungs, and intestine tissues. In vitro, CKoV infection of HEK293T cells triggers apoptosis, with the viral structural protein VP1 identified as sufficient to induce apoptosis. VP1 activates the mitochondrial (intrinsic) apoptotic pathway, as evidenced by increased reactive oxygen species (ROS) production, activation of caspase-9 and caspase-3, elevated Bax/Bcl-2 ratio, cytochrome c release, and Bax translocation to mitochondria. Notably, VP1 associates with the pro-apoptotic protein BAD (BCL2-associated agonist of cell death) and co-localizes with it in mitochondria. Overexpression of BAD enhances VP1-induced apoptosis, whereas BAD knockdown by siRNA suppresses it, demonstrating that BAD plays a contributory role in VP1-induced apoptosis. These findings uncover a novel VP1–BAD mitochondrial apoptosis axis in CKoV pathogenesis and suggest that VP1 may merit further investigation as a potential molecular target for antiviral intervention against CKoV-associated enteritis in goats.

## Simple Summary

Caprine kobuvirus (CKoV) is a virus that causes diarrhea in young goats, leading to significant economic losses for goat farmers. However, it is not fully understood how this virus causes disease. In this study, we investigated the molecular mechanisms by which CKoV induces cell death. We found that CKoV infection triggers apoptosis (programmed cell death) in goat tissues and in cultured cells. Importantly, we identified a specific viral protein, VP1, as sufficient to induce this effect. VP1 acts by interacting with a host cell protein called BAD (BCL2-associated agonist of cell death), which in turn activates the mitochondrial (intrinsic) apoptotic pathway, a well-known cell death signaling cascade. This process involves a series of molecular events, including the production of reactive oxygen species, activation of specific cell-death enzymes (caspases), and changes in mitochondrial membrane permeability. Our findings reveal a previously unknown mechanism by which CKoV causes disease and identify VP1 as a potential target for the development of antiviral strategies to control CKoV-associated diarrhea in goats.

## 1. Introduction

Kobuvirus (KoV), a genus within the *Picornaviridae* family [1], is known to cause gastrointestinal inflammation in both humans and animals, including cattle and pigs [2,3,4]. It is closely associated with diarrhea in various species, such as dogs, cats, and goats. The virus is globally distributed, posing significant threats to both public health and the livestock industry [5,6,7]. Caprine kobuvirus (CKoV) has been detected in goats in four countries [6,8,9,10,11]. In 2019, our team identified CKoV in goats in southwestern China and in Tibetan sheep on the Qinghai–Tibet Plateau through metagenomic sequencing, suggesting that CKoV was already widespread in these regions [6,12]. Subsequent studies further revealed that CKoV is also prevalent in goat herds in Sichuan Province, with detection rates of 77.8% and 55.74% in diarrheic kids from two independent studies, respectively [6,13]. In 2020, CKoV was first isolated from diarrheic lambs in China, causing severe watery diarrhea, systemic infection, and multi-organ damage, with morbidity reaching 100% [6]. These findings confirm that CKoV is an emerging diarrheal pathogen in goat kids in China and highlight the need to elucidate its pathogenic mechanisms.

Despite its emerging importance, research on CKoV is still relatively limited, with most studies focusing on its genome and epidemiology [8,9,10]. However, its pathogenic mechanisms, particularly the role of apoptosis in disease progression, remain poorly understood. Apoptosis, a key pathogenic mechanism employed by many picornaviruses, facilitates viral replication and dissemination. For instance, coxsackievirus (CV), poliovirus (PV), enterovirus (EV), Seneca virus (SV), and foot-and-mouth disease virus (FMDV) have been shown to manipulate host cell survival and immune evasion through apoptosis [14,15,16,17].

The structural protein VP1 of picornaviruses is a multifunctional protein that is critical for viral replication and cytotoxicity [3,18,19]. It has been demonstrated to play a pivotal role in virus-induced apoptosis [20,21]. For example, the VP1 protein of FMDV activates apoptosis pathways in cancer cells through phosphatidylinositol 3-kinase stimulation and induces apoptosis via the AKT pathway in BHK-21 cells, regulated by the TP53 protein. Similarly, VP1 from SV interacts with the pro-apoptotic protein BAD (BCL2-associated agonist of cell death), triggering the mitochondrial apoptotic pathway, which involves upregulation of BAX and BAD from the Bcl-2 family and cleavage of caspase-3, leading to significant apoptosis in HEK293T cells.

However, the mechanisms of CKoV-induced cell death, particularly those involving the VP1 protein, remain unclear. In this study, we investigated apoptosis induced by CKoV infection both in vivo and in vitro, with a particular focus on the role of the VP1 protein in HEK293T cells as a model system for mechanistic exploration. Our findings indicate that VP1 interacts with the pro-apoptotic protein BAD to induce apoptosis via the mitochondrial pathway, providing novel insights into the pathogenic mechanisms of CKoV.

## 2. Material and Methods

### 2.1. Cells and Virus

Vero cells (ATCC^®^ CCL-81™) and HEK293T cells (ATCC^®^ CRL-3216™) were obtained from the American Type Culture Collection (ATCC, Manassas, VA, USA). These cells were cultured in Dulbecco’s Modified Eagle’s Medium (DMEM) supplemented with 10% heat-inactivated fetal bovine serum (TransGen Biotech, Beijing, China) and maintained under a 5% CO_2_ atmosphere at 37 °C. The caprine kobuvirus (CKoV) strain was isolated from goat diarrhea samples and purified via plaque assay. The virus was propagated in Vero cells at 37 °C. After 72 h of infection, the cultures underwent three freeze–thaw cycles, followed by centrifugation at 1500 rpm for 10 min to collect the supernatant, which was subsequently stored at −80 °C for further experiments.

### 2.2. Antibodies

The following primary antibodies were used in the study: anti-β-actin (AC026) from ABclonal (Wuhan, China); anti-caspase-3 (222119), anti-caspase-9 (66169), anti-caspase-8 (222121), anti-caspase-12 (160136), anti-BAD (380970), anti-Bax (380709), anti-PARP1 (380451), anti-cleaved PARP1 (380374), anti-cleaved caspase-3 p17 (341034), anti-cleaved caspase-9 (340263), anti-cleaved caspase-8 (250160), anti-cytochrome C (R22667) and anti-Bcl-2 (381702) from Zen-bioscience (Chengdu, China). Mouse and rabbit polyclonal antibodies against CKoV VP1 were prepared in our laboratory. The secondary antibodies included AffiniPure Goat Anti-Mouse IgG (H+L) (BA1038) and AffiniPure Goat Anti-Rabbit IgG (H+L) (BA1039) from Boster Biological Technology (Wuhan, China), and goat anti-mouse IgG (H+L) Texas Red (BS10002), goat anti-rabbit IgG (H+L) Texas Red (BS10003) from Bioworld (Nanjing, China).

### 2.3. Plasmid Constructs and Transfection

For the construction of the VP1 expression plasmid, viral RNA from CKoV was extracted, amplified, and cloned into the pcDNA3.1(+) vector using *Hind* III and *Xho* I restriction sites to generate pcDNA3.1-VP1. The BAD expression plasmid was similarly constructed from HEK293T cells and cloned into the pCMV vector. HEK293T cells were transfected with either the VP1 or BAD plasmids using X-tremeGENE HP DNA transfection reagent (Roche, Basel, Switzerland) according to the manufacturer’s protocol. Untransfected cells and cells transfected with empty vectors were used as controls. For BAD knockdown, a specific siRNA targeting human BAD was designed based on a previously validated sequence: 5′-AAGAAGGGACTTCCTCGCCCG-3′. The siRNA and a negative control siRNA (NC) were synthesized by GenePharma Co., Ltd. (Shanghai, China). Both siRNAs were dissolved in DEPC-treated water at a final concentration of 20 μM. HEK293T cells were transfected with BAD siRNA or NC siRNA using jetPRIME transfection reagent (Polyplus-transfection SA, Illkirch, France) according to the manufacturer’s instructions.

### 2.4. In Vivo Experiments

Healthy 7-day-old baby goats, confirmed negative for CKoV by RT-qPCR and negative for CKoV-specific neutralizing antibodies (titer < 1:2), were obtained from a farm in Sichuan Province. Two goats (*n* = 2) were orally inoculated with 2 mL of medium containing 1 × 10^6^·^7^ TCID_50_ of CKoV, while two control goats (*n* = 2) received the same medium without the virus. This pilot study was designed to provide initial evidence for CKoV-induced apoptosis in vivo. Post-infection, the goats were monitored for clinical symptoms. At 45 h post-infection, all animals were euthanized in accordance with the guidelines of the Ethical Committee of the Institute (SMU-202501088), and tissue samples from the spleen, lungs, and intestinal tissues were collected, fixed in 10% neutral buffered formalin, embedded in paraffin wax, and analyzed for apoptosis using the TUNEL assay. Tissue sections were dewaxed, rehydrated, and treated for quenching of endogenous peroxidase activity. The TUNEL analysis was carried out for the histological detection of apoptotic cells using a TUNEL apoptosis detection kit. Tissue sections from the control group were used as negative controls.

### 2.5. Fluorescence Microscopy

HEK293T cells transfected with the VP1 plasmid were analyzed for VP1 expression using immunofluorescence. Briefly, at 48 h post transfection, cells were washed three times with ice-cold phosphate-buffered saline (PBS), fixed with 4% paraformaldehyde (PFA), and permeabilized with 0.3% Triton X-100. Primary antibodies specific to VP1, caspase-3, and caspase-9 were applied, followed by incubation with fluorochrome-conjugated secondary antibodies at room temperature for 1 h. Fluorescence signals were observed using a fluorescence microscopy (TOKYO OHKA KOGYO CO., LTD., Tokyo, Japan).

### 2.6. Flow Cytometry Analysis

Apoptosis was measured using an annexin V-fluorescein isothiocyanate (FITC) apoptosis detection kit (Beijing 4A Biotech Co., Ltd., Beijing, China) according to the manufacturer’s instructions. Cells were harvested at 24 and 48 h post-infection with CKoV or post-transfection with VP1. After trypsinization (without EDTA), the cells were washed with ice-cold PBS and centrifuged. The cell pellets were resuspended in 500 μL of binding buffer containing 5 μL of annexin V-FITC and 10 μL of propidium iodide (PI). Following a 20-min incubation at room temperature in the dark, the samples were analyzed on a flow cytometer (CytoFLEX, Beckman Coulter, Brea, CA, USA) within 60 min. For each sample, 10,000 events were acquired, and the gating strategy was set based on forward scatter (FSC) and side scatter (SSC) to exclude debris and doublets. Compensation was performed using single-stained controls (annexin V-FITC only and PI only). Unstained cells were used as negative controls.

### 2.7. Reactive Oxygen Species (ROS) Detection

The intracellular production of ROS in VP1-transfected HEK293T cells was quantified using a fluorometric ROS detection kit (MAK143; Sigma-Aldrich, St. Louis, MO, USA) according to the manufacturer’s instructions. Dichlorofluorescin diacetate (DCFH-DA) was added to the cells and incubated for 20 min at 37 °C. After centrifugation at 350 rpm for 5 min, the supernatant was aspirated for detection and analysis. Fluorescence signals were measured using a flow cytometer (CytoFLEX, Beckman Coulter, Brea, CA, USA).

### 2.8. Detection of Caspase Activation

Caspase-3 and caspase-9 activation was assessed by immunofluorescence and Western blotting. Cells were harvested at various time points post-transfection, and cleaved (activated) caspase-3 and caspase-9 were detected using specific antibodies against their cleaved forms. For immunofluorescence, cells were fixed, permeabilized, and incubated with primary antibodies specific to cleaved caspase-3 and cleaved caspase-9, followed by incubation with Texas Red-conjugated secondary antibodies. Fluorescence signals were visualized under a fluorescence microscope. For Western blotting, cell lysates were separated by SDS-PAGE and probed with the same antibodies. Signal detection was performed using an ECL kit (Thermo Fisher Scientific, Waltham, MA, USA).

### 2.9. Western Blotting and Cytosolic Fractionation

HEK293T cells were lysed using NP-40 lysis buffer, and protein extracts were separated by SDS-PAGE and transferred onto PVDF membranes. Membranes were blocked with 5% skim milk and incubated overnight with primary antibodies at 4 °C, followed by incubation with HRP-conjugated secondary antibodies. Signal detection was performed using an ECL detection kit. To examine mitochondrial protein release, mitochondrial and cytosolic fractions were prepared using a mitochondrion isolation kit (Beyotime, Shanghai, China), and proteins were analyzed by Western blotting. To analyze mitochondrial protein release, VP1-transfected cell pellets were washed with cold PBS and isolated using a mitochondrion isolation kit (C3601; Beyotime, Shanghai, China). Briefly, cells were lysed by passing through a 26-gauge needle, and after centrifugation, the cytosolic supernatant and mitochondrial pellet were collected. Protein concentrations were measured with a BCA protein assay kit (23225; Thermo Fisher Scientific), and samples were separated by SDS-PAGE for Western blot analysis.

### 2.10. Molecular Docking and Co-Immunoprecipitation

The VP1 structure was generated by homology modeling using the SWISS-MODEL server based on the experimental structure of human Aichivirus capsid protein VP1 (PDB ID: 5GKA, chain A) as template. The BAD structure was retrieved from the AlphaFold Protein Structure Database (AF-Q92934-F1). Protein–protein docking was performed using the HDOCK server in template-free global blind docking mode, with a 15° rotational sampling interval and a 1.2 Å translational grid spacing. The HDOCK docking score for the Rank 1 VP1–BAD complex was −341.28. Binding affinity was further predicted using PRODIGY, yielding a ΔG of −11.7 kcal/mol and a Kd of 5.6 × 10^−9^ M at 310 K. The final complex was visualized using PyMOL (version 1.3, Schrödinger, LLC, New York, NY, USA). Co-immunoprecipitation experiments were performed by co-transfecting HEK293T cells with pcDNA3.1-VP1 and pCMV-BAD plasmids, followed by lysis and incubation with anti-VP1 or anti-BAD antibodies. Immunoprecipitated protein complexes were analyzed by Western blotting.

### 2.11. Laser Confocal Microscopy

Co-localization of VP1 and BAD proteins in HEK293T cells was observed using laser confocal microscopy. Cells were transfected with pcDNA3.1-VP1, fixed with ice-cold acetone, and stained with primary and secondary antibodies specific to VP1 and BAD. Fluorescent signals were visualized using a laser confocal microscope.

### 2.12. Statistical Analysis

All experiments were performed in triplicate (independent biological replicates). Statistical analyses were carried out using GraphPad Prism 6 software. For comparisons between two groups, Student’s *t*-test was used. For comparisons involving multiple groups (e.g., different MOIs, time points, or transfection conditions), one-way or two-way analysis of variance (ANOVA) was applied, followed by Tukey’s post hoc test for multiple comparisons. A *p*-value of <0.05 was considered statistically significant. All data are presented as mean ± standard deviation (SD).

### 2.13. Quantitative Real-Time PCR (qPCR)

Total RNA was extracted from cultured cells using RNAios Plus (TaKaRa Bio Inc., Kusatsu, Japan) according to the manufacturer’s instructions. Reverse transcription was performed using the PrimeScript RT Reagent Kit (TaKaRa Bio Inc., Kusatsu, Japan) with random hexamer primers. qPCR was carried out using ChamQ Universal SYBR Green qPCR Master Mix (Vazyme, Nanjing, China) on a CFX96 Real-Time PCR System (Bio-Rad, Hercules, CA, USA). The cycling conditions were as follows: initial denaturation at 95 °C for 30 s, followed by 40 cycles of denaturation at 95 °C for 5 s and annealing/extension at 60 °C for 30 s. The primer sequences used for amplification of target genes and the internal reference gene (**GAPDH**) are listed in Supplementary Table S1. Amplification efficiencies for all primer pairs were determined using standard curves generated from serial dilutions of cDNA and were found to be between 90% and 110%. Relative expression levels were calculated using the 2^−Δ^^ΔCt^ method. All reactions were performed in triplicate.

## 3. Results

### 3.1. CKoV Infection Induced Apoptosis in Various Tissues of Infected Goats

**Figure 1:**
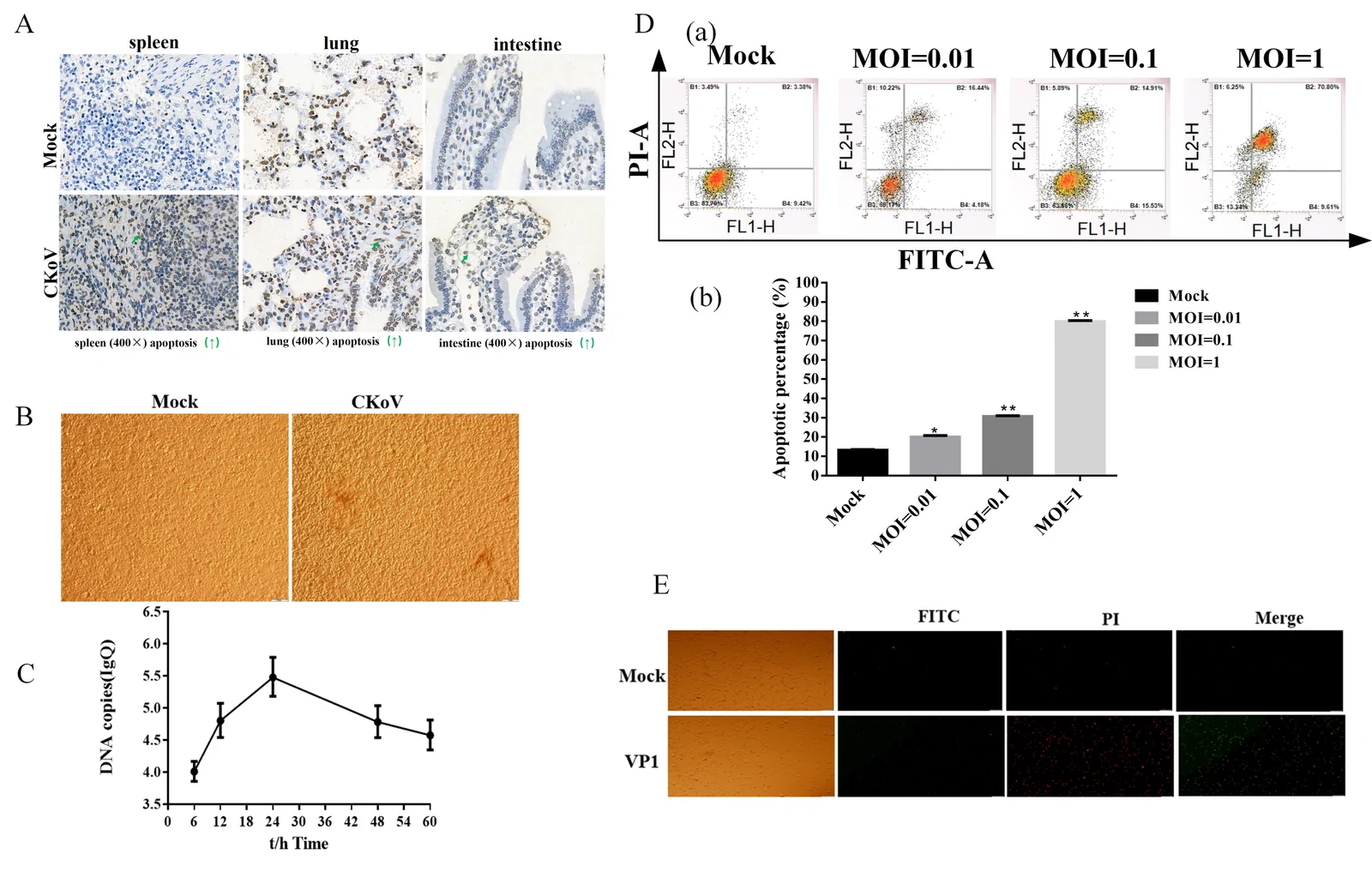
CKoV Infection Induces Apoptosis. (**A**) TUNEL staining of spleen, lung, and intestinal tissues from CKoV-infected goats and control goats. TUNEL-positive cells are visualized as brown staining. (**B**) Representative images showing cytopathic effects (CPE) in HEK293T cells 24 h post-infection with CKoV (MOI = 0.1). (**C**) Viral RNA quantification in HEK293T cells at different time points post-infection. (**D**) Flow cytometry and microscopy analysis of apoptosis in HEK293T cells infected with CKoV for 24 h at MOIs of 0.01, 0.1, and 1. (**a**) Cells were stained with Annexin V-FITC and propidium iodide (PI) to differentiate early apoptosis (Annexin V-positive/PI-negative) and late apoptosis (Annexin V-positive/PI-positive). (**b**) Data are presented as mean ± SD from three independent experiments. *, *p* < 0.05; **, *p* < 0.01. (**E**) Representative fluorescence microscopy images showing the fluorescence intensity of Annexin V and PI staining.

Two goat kids infected with CKoV developed watery diarrhea at 24 and 30 h post-infection, followed by bloody stools and a temperature rise to 42 °C by 36 h. Severe dehydration and an inability to stand were observed at 40 h post-infection, while no abnormalities were seen in the control group. To examine the effect of CKoV on tissue apoptosis, we performed TUNEL staining on spleen, lung, and intestinal sections from infected and control goats. TUNEL-positive cells appeared more frequently in tissues from infected animals than in those from controls. These findings suggest that CKoV infection may induce apoptosis in multiple organs, but due to the limited sample size (*n* = 2 per group), further studies with larger cohorts are needed to confirm these observations (Figure 1A).

### 3.2. CKoV Induced Apoptosis in HEK293T Cells

Previous studies have shown that CKoV replication induced shrinkage, shedding, and fusion in infected cells [22]. To determine whether CKoV infection leads to apoptosis in permissive HEK293T cells, we inoculated the cells with CKoV at multiplicities of infection (MOIs) of 0.01, 0.1, and 1. Cytopathic effects, such as cell shrinkage, detachment, and fusion, were observed in infected cells compared to uninfected controls (Figure 1B). The replication efficiency of CKoV was highest 24 h post-infection, as confirmed by quantification of viral RNA (Figure 1C). Flow cytometry analysis using Annexin V-FITC/PI double staining demonstrated a significant increase in both early and late apoptosis in HEK293T cells infected with CKoV at an MOI of 0.1 (*p* < 0.05) (Figure 1D,E).

### 3.3. VP1 Induces Apoptosis via a Mitochondrion-Mediated Pathway

**Figure 2:**
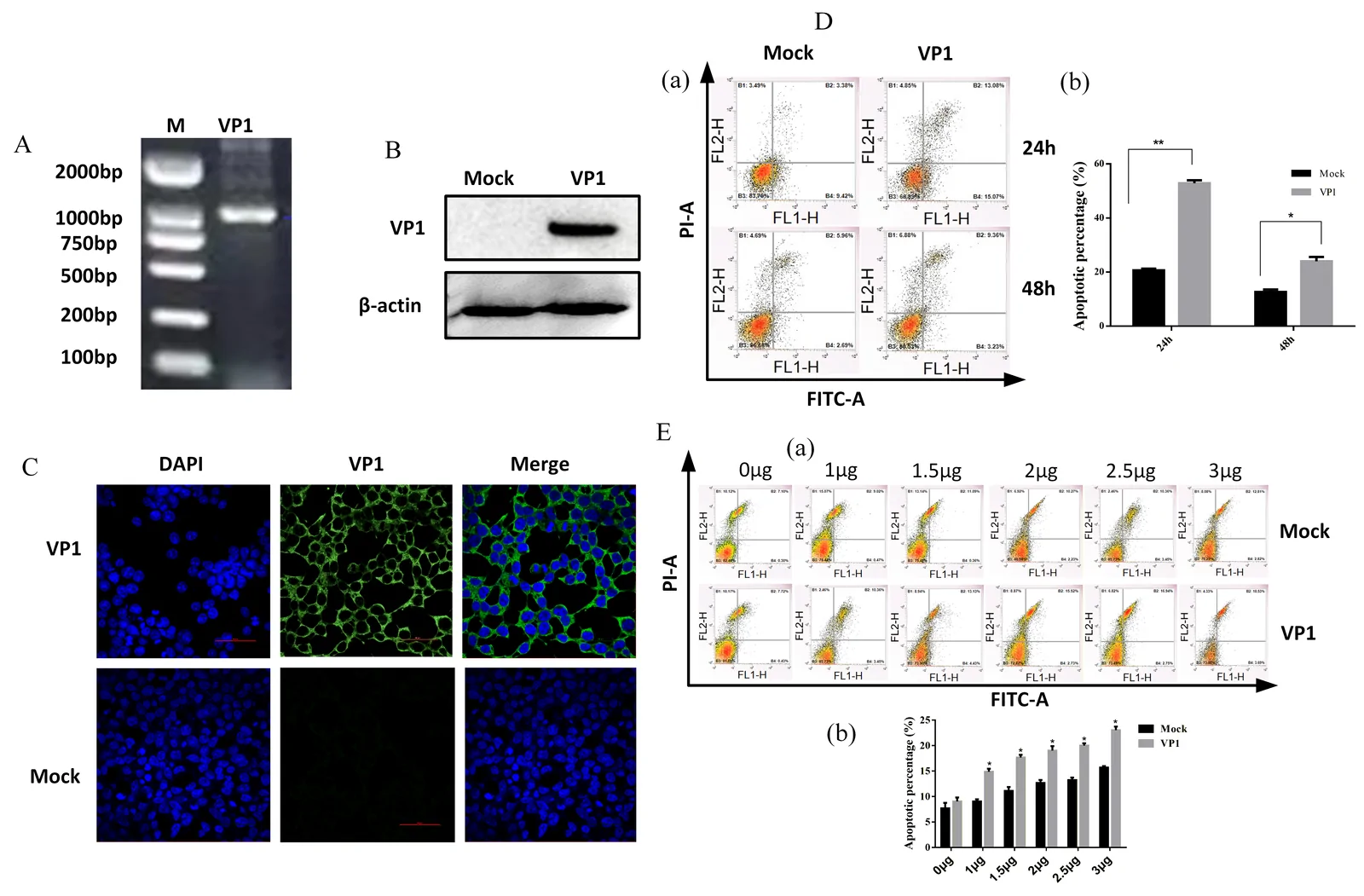
VP1-Induced Apoptosis in HEK293T Cells. (**A**) PCR analysis confirming successful transfection of the VP1 plasmid into HEK293T cells. (**B**) Western blot showing VP1 protein expression in transfected HEK293T cells. (**C**) Immunofluorescence assay (IFA) confirming VP1 expression in transfected cells. (**D**) Representative flow cytometry plots showing VP1-induced apoptosis in HEK293T cells at 24 h and 48 h post-transfection. (**E**) Quantification of apoptosis showing a dose-dependent relationship between VP1 expression and cell death. Data are presented as mean ± SD from three independent experiments. *, *p* < 0.05; **, *p* < 0.01; these values are statistically significantly different from those of the control group.

HEK293T cells transfected with the CKoV VP1 plasmid (Figure 2A–C) exhibited morphological changes characteristic of apoptosis at 24 h post-transfection, with the number of apoptotic cells increasing in a dose-dependent manner (Figure 2D,E). These results suggest that VP1 is sufficient to induce apoptosis under the tested conditions.

**Figure 3:**
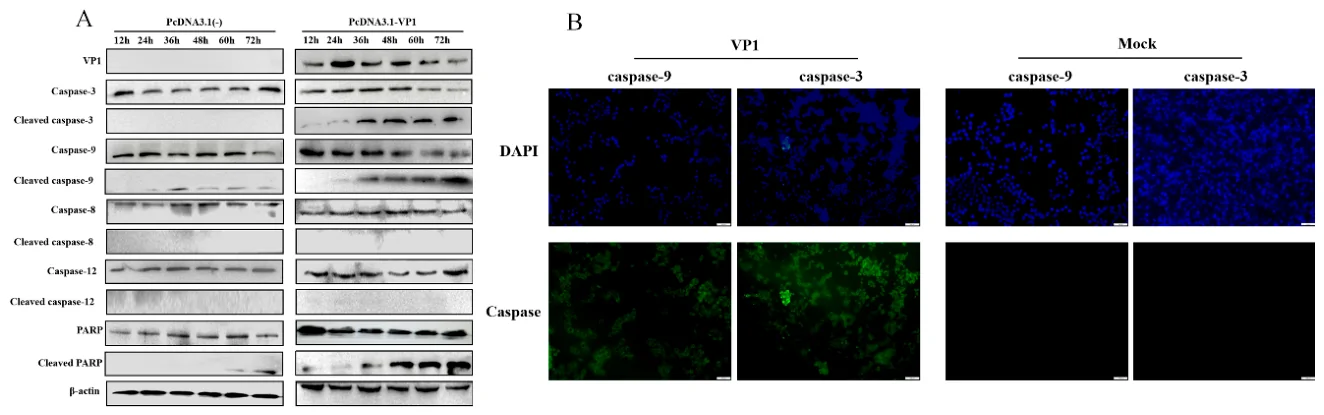
Activation of Caspase-9 and Caspase-3 by VP1 in HEK293T Cells. (**A**) Western blot analysis of cleaved caspase-9, caspase-3, and PARP in HEK293T cells at various time points post-transfection. β-Actin was used as a loading control. (**B**) Immunofluorescence staining of active caspase-9 and caspase-3 in HEK293T cells transfected with VP1 or mock-transfected (control). Green fluorescence represents active caspases.

The involvement of the mitochondrial pathway in VP1-induced apoptosis was further examined by measuring caspase-9 and caspase-3 activities in transfected cells. Immunofluorescence revealed activation of both caspases at 36 h post-transfection (Figure 3B), which was confirmed by Western blot analysis of cleaved caspase-9 and caspase-3, along with poly (ADP-ribose) polymerase (PARP) (Figure 3A).

**Figure 4:**
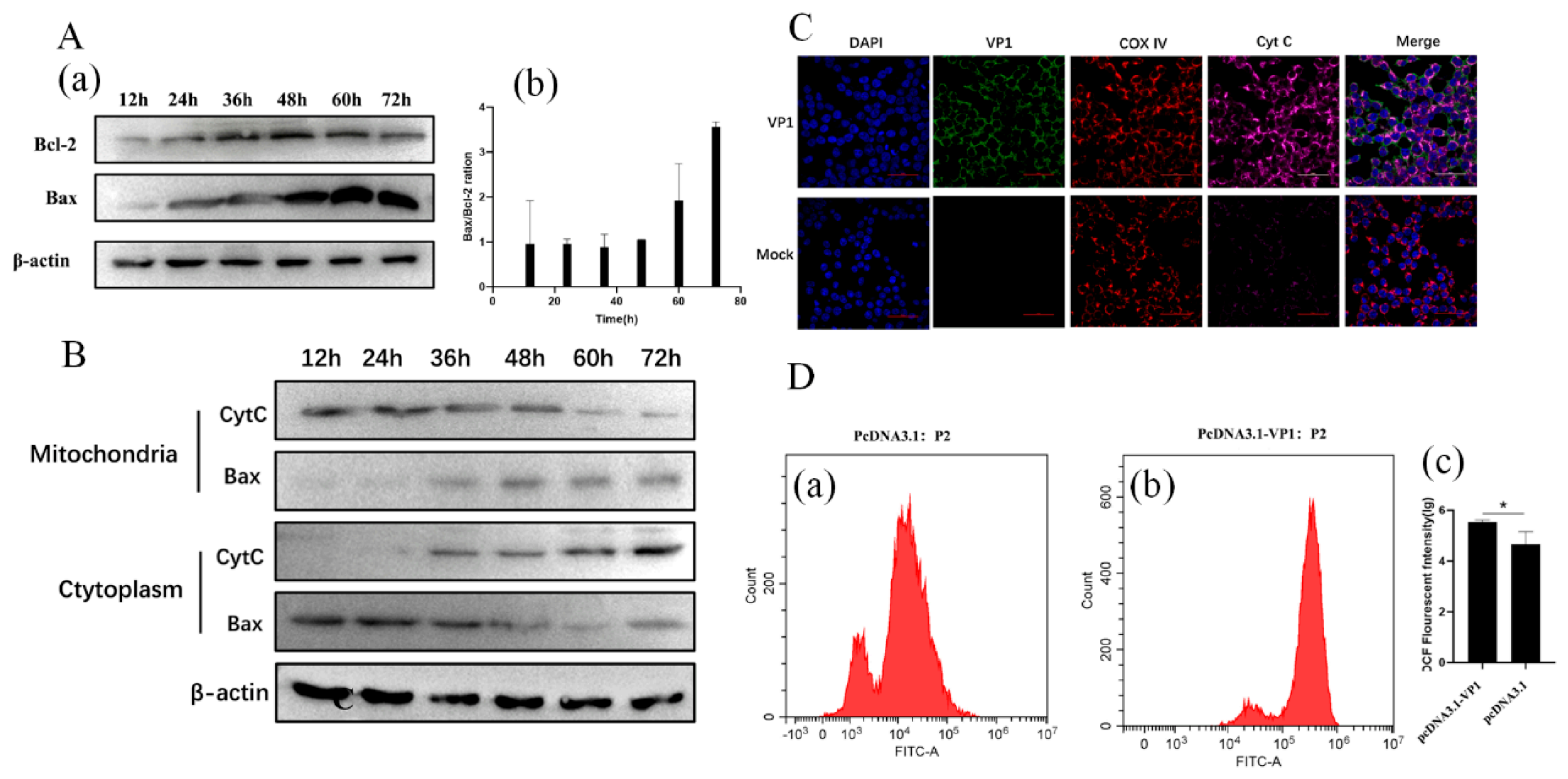
VP1 Activates the Mitochondrial Apoptotic Pathway via Bax Translocation and Cytochrome C Release. (**A**) Western blot analysis of Bax expression (**a**) and Bax/Bcl-2 ratio (**b**) in VP1-transfected HEK293T cells at various time points post-transfection. (**B**) Western blot analysis of Bax and cytochrome C (Cyt c) translocation. (**C**) Immunofluorescence confirms the cytosolic release of Cyt c in VP1-transfected cells. (**D**) Measurement of intracellular reactive oxygen species (ROS) levels in HEK293T cells. (**a**) ROS levels in cells transfected with empty vector (pcDNA3.1). (**b**) ROS levels in cells transfected with pcDNA3.1-VP1. (**c**) Quantification of ROS levels.

Additionally, VP1 significantly upregulated Bax expression and the Bax/Bcl-2 ratio (Figure 4A), promoting the translocation of Bax to the mitochondria and the release of cytochrome C into the cytoplasm (Figure 4B,C). Under our experimental conditions, we did not observe detectable activation of caspase-8 or caspase-12. We therefore focus our study on the mitochondrial pathway. Furthermore, intracellular ROS production was significantly elevated in VP1-transfected cells, suggesting an association between VP1 expression and increased ROS production under the tested conditions (Figure 4D).

### 3.4. VP1 Protein Interacts with the Pro-Apoptotic Protein BAD

**Figure 5:**
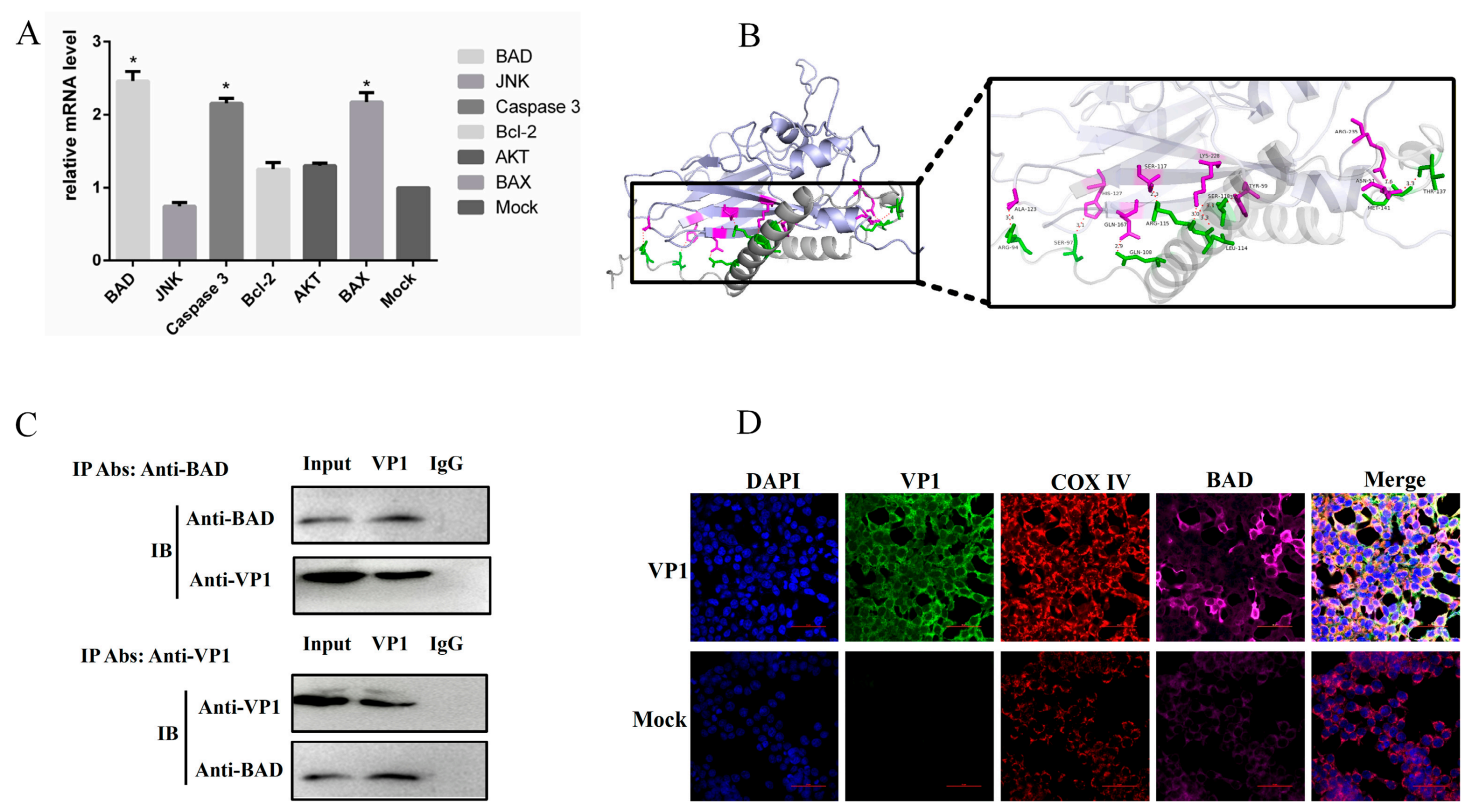
VP1 Protein Interacts with Pro-Apoptotic Protein BAD in HEK293T Cells. (**A**) Quantitative PCR analysis of BAD, JNK, Caspase 3, Bcl-2, AKT, and Bax mRNA levels in HEK293T cells transfected with VP1. (**B**) Molecular docking analysis of the VP1–BAD interaction. VP1 is shown as a light gray cartoon, and BAD as a light purple cartoon. Hydrogen-bonding residues of VP1 are highlighted in pink, and those of BAD in green. Hydrogen bonds between VP1 and BAD are indicated by red dashed lines, with distances labeled in Ångströms. The VP1 structure was generated by homology modeling using SWISS-MODEL based on PDB ID 5GKA, and the BAD structure was obtained from the AlphaFold Database (AF-Q92934-F1). Docking was performed using HDOCK (score: −341.28), and binding affinity was predicted by PRODIGY (ΔG = −11.7 kcal/mol, Kd = 5.6 × 10^−9^ M at 310 K). Residues involved in hydrogen bonding include VP1 residues ALA123, HIS127, SER117, GLN167, LYS228, TYR59, ASN51, ARG235 and BAD residues ARG94, SER97, ARG115, GLN108, SER118, LEU114, THR137, MET141, with distances ranging from 1.6 to 3.4 Å. (**C**) Co-immunoprecipitation assay of VP1 and BAD in HEK293T cells. (**D**) Confocal microscopy images of VP1 (green) and BAD (pink) localization in mitochondria of transfected HEK293T cells.

To investigate the role of the pro-apoptotic protein BAD in VP1-induced apoptosis, we first examined the mRNA levels of BAD, Bax, and caspase-3 in VP1-transfected HEK293T cells. BAD was significantly upregulated 24 h post-transfection (Figure 5A). Using co-immunoprecipitation assays and molecular docking, we found that VP1 associates with BAD. Molecular docking predicted hydrogen bonding between VP1 residues (e.g., ALA123, HIS127, SER117, GLN167, LYS228, TYR59, ASN51, ARG235) and BAD residues (e.g., ARG94, SER97, ARG115, GLN108, SER118, LEU114, THR137, MET141), with distances ranging from 1.6 to 3.4 Å (Figure 5B,C). Confocal microscopy further demonstrated the co-localization of VP1 and BAD in the mitochondria (Figure 5D).

### 3.5. BAD Plays a Contributory Role in VP1-Induced Apoptosis

**Figure 6:**
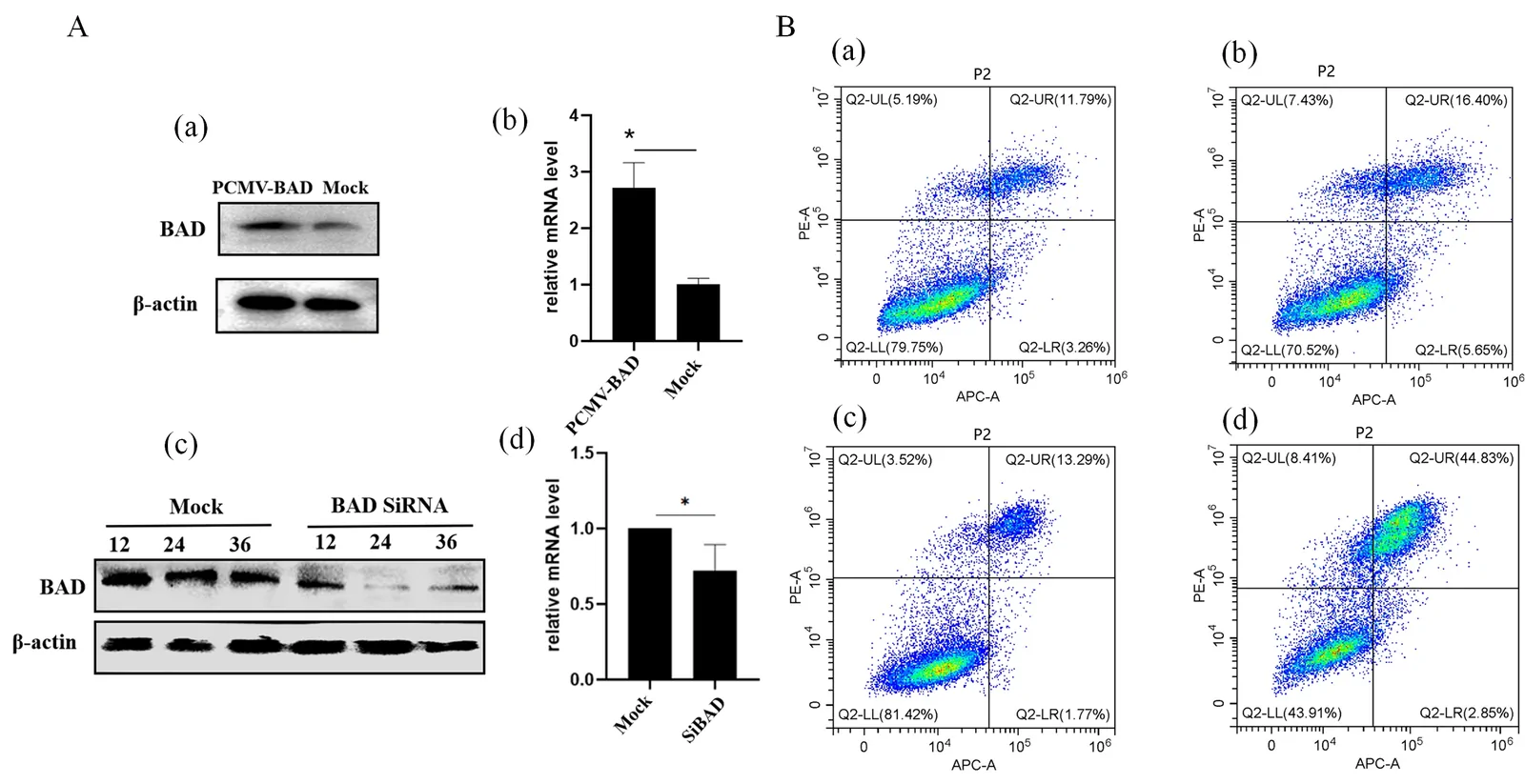
BAD Enhances VP1-Induced Apoptosis in HEK293T Cells. (**A**) BAD overexpression and knockdown in HEK293T cells. (**a**) Western blot analysis of BAD expression after transfection with BAD overexpression plasmid. (**b**) Relative BAD mRNA levels quantified by qPCR under overexpression conditions. (**c**) Western blot analysis of BAD expression after transfection with BAD siRNA (knockdown). (**d**) Relative BAD mRNA levels quantified by qPCR under knockdown conditions. (**B**) Flow cytometry analysis of apoptosis in HEK293T cells transfected with different combinations of plasmids: (**a**) negative control (empty vector pcDNA3.1); (**b**) VP1-only transfection; (**c**) VP1 co-transfected with BAD siRNA (knockdown); (**d**) VP1 co-transfected with BAD (overexpression).

To assess whether VP1-induced apoptosis is dependent on BAD, we generated HEK293T cell models with BAD overexpression and BAD knockdown via siRNA (Figure 6A). Flow cytometry analysis showed that BAD overexpression significantly enhanced VP1-induced apoptosis, increasing the apoptotic rate from 22.05% (in VP1-only transfected cells) to 47.68% (in VP1 and BAD co-transfected cells) (Figure 6B). In contrast, BAD knockdown reduced VP1-induced apoptosis to 15.06%. These findings suggest that BAD plays a contributory role in VP1-induced mitochondrial dysfunction and apoptosis.

## 4. Discussion

The VP1 protein of picornaviruses is widely known for its ability to mediate cytotoxicity and apoptosis in infected cells, contributing significantly to viral pathogenesis [3,18]. In this study, we aimed to elucidate the mechanisms by which caprine kobuvirus (CKoV) induces apoptosis, particularly focusing on the role of the viral structural protein VP1. Our findings suggest that CKoV infection induces apoptosis both in vivo and in vitro, and that the VP1 protein is sufficient to trigger this process through the mitochondrial apoptotic pathway under the experimental conditions tested.

Apoptosis is a well-recognized pathogenic mechanism used by picornaviruses to facilitate viral replication and transmission by manipulating host cell death [14,15,16,17,23]. Previous studies on other picornaviruses, such as FMDV and SV, have highlighted the importance of VP1 in inducing apoptosis via interactions with the host’s apoptotic machinery [20]. Three major apoptotic pathways are commonly activated in response to cellular stress: the mitochondrial-mediated pathway, the endoplasmic reticulum (ER) stress-induced pathway, and the death receptor-mediated pathway [24]. Our study clearly demonstrated that VP1 of CKoV primarily activates the mitochondrial pathway. This was confirmed by the upregulation of Bax, the increased Bax/Bcl-2 ratio, and the release of cytochrome C from the mitochondria into the cytoplasm, leading to the activation of caspase-9 and caspase-3. We did not observe significant activation of caspase-8 or caspase-12, and we therefore focus our study on the mitochondrial pathway.

The mitochondrial apoptotic pathway is tightly regulated by the Bcl-2 family of proteins, which includes both pro-apoptotic members like Bax and BAD, and anti-apoptotic members like Bcl-2 [25]. Our results demonstrated that VP1 significantly upregulated BAD expression, and molecular docking predictions together with co-immunoprecipitation experiments indicated an interaction between VP1 and BAD. This interaction was found to contribute to the apoptotic process, as BAD overexpression significantly enhanced VP1-induced apoptosis, while BAD knockdown via siRNA reduced apoptosis. These findings suggest that BAD contributes to VP1-induced mitochondrial dysfunction and apoptosis.

One novel aspect of our study is the identification of ROS as an important factor in VP1-induced apoptosis. ROS are primarily generated in the mitochondria, and their excessive production can lead to oxidative stress and the activation of apoptosis. We observed a significant increase in ROS levels in VP1-transfected cells, which likely contributed to the disruption of mitochondrial integrity and the subsequent release of cytochrome C. While the exact mechanism of ROS production in response to VP1 remains to be elucidated, our data suggest an association between VP1 expression and increased ROS production, although further experimental validation would be needed to establish a causal relationship.

Our findings also align with previous studies on other picornaviruses, such as FMDV and SV [20,21], which have demonstrated that VP1 is a multifunctional protein capable of inducing apoptosis via various pathways. For example, FMDV VP1 has been shown to activate the AKT apoptotic pathway and promote apoptosis through TP53 regulation in cancer cells. Similarly, SV VP1 induces apoptosis by interacting with BAD and activating the mitochondrial pathway [26]. However, it is important to note that the VP1–BAD interaction experiments were performed in an overexpression model rather than during authentic viral infection. Therefore, our findings demonstrate VP1 sufficiency in the experimental model, but further studies would be required to establish VP1 necessity during CKoV infection. In addition, it should be noted that the present cell-based experiments were performed in HEK293T cells, a human embryonic kidney-derived cell line, which was chosen for its high transfection efficiency and well-characterized apoptotic machinery. While this model facilitates mechanistic studies, caution is needed when extrapolating these findings to CKoV-associated enteric pathogenesis in goats, and future validation using caprine-derived cells or intestinal organoids is warranted. In addition, increased viral RNA in infected cells does not by itself demonstrate production of infectious progeny; viral titers were not quantified in this study. Furthermore, the purity of the mitochondrial and cytosolic fractions was not verified using compartment-specific marker proteins, which represents a limitation of our fractionation experiments. Nevertheless, the observed Bax translocation and cytochrome c release were consistently detected under our experimental conditions. Importantly, these biochemical fractionation results are supported by complementary immunofluorescence imaging data, which clearly demonstrated the mitochondrial localization of VP1, its co-localization with BAD, and the release of cytochrome c into the cytosol. Taken together, the combined evidence from both fractionation and imaging approaches supports our conclusions regarding mitochondrial involvement in VP1-induced apoptosis.

## 5. Conclusions

In conclusion, this study provides important insights into the molecular mechanisms of CKoV-induced apoptosis, with a particular focus on the VP1 protein. Our findings indicate that VP1 induces apoptosis via the mitochondrial pathway through its interaction with BAD, leading to mitochondrial dysfunction, reactive oxygen species (ROS) production, and activation of the caspase cascade. These findings advance our understanding of CKoV pathogenesis and suggest that VP1-induced apoptotic pathways may merit further investigation as potential targets for the development of novel antiviral strategies, although their therapeutic relevance remains to be evaluated in future studies.

## Data Availability

The original contributions presented in this study are included in the article. Further inquiries can be directed to the corresponding authors.

## Supplementary Materials

The following supporting information can be downloaded at: https://www.mdpi.com/article/10.3390/vetsci13090993/s1 File S1: Original Western blot images; Table S1: Primers used in this study.

## Abbreviations

The following abbreviations are used in this manuscript:

CKoV	Caprine kobuvirus
KoV	Kobuvirus
ROS	Reactive oxygen species
HEK293T	Human embryonic kidney 293T cells
FITC	Fluorescein isothiocyanate
BAD	BCL2-associated agonist of cell death
