# Supplementary material for: Caprine Kobuvirus VP1 Protein Activates the Mitochondrial Apoptotic Pathway via Interaction with BAD
Source: Vet Sci. 2026 Sep 19;13(9):993. doi: 10.3390/vetsci13090993 (PMC13612080; doi:10.3390/vetsci13090993)

Figure 2
VP1

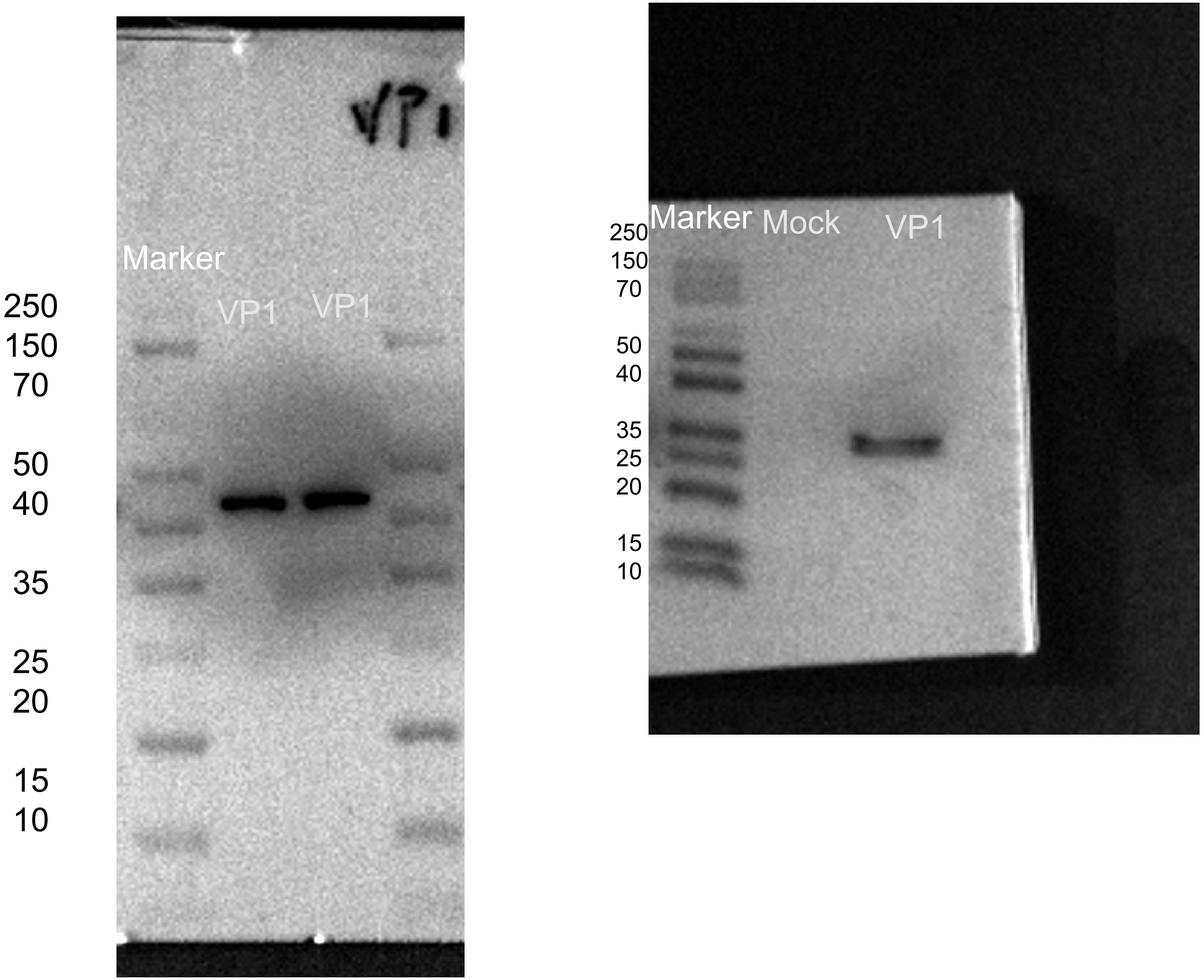
Marker
VP1
VP1
250
150
70
50
40
35
25
20
15
10
VP1
Marker
Mock
VP1
250
150
70
50
40
35
25
20
15
10

Figure 3
Procaspase-3 in empty vector–transfected cells 32kDa

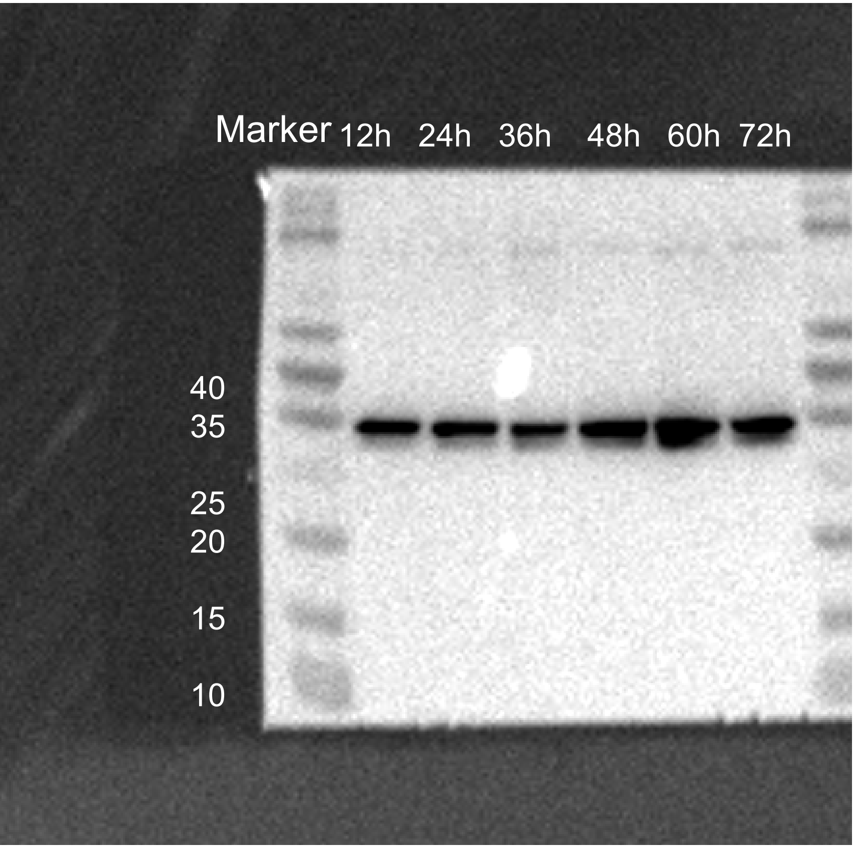

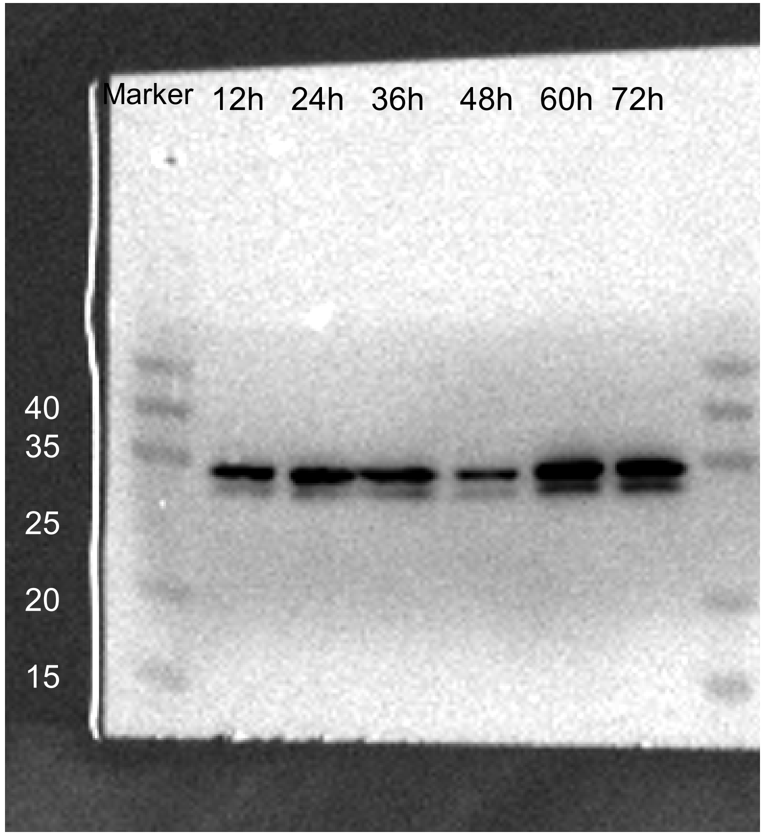

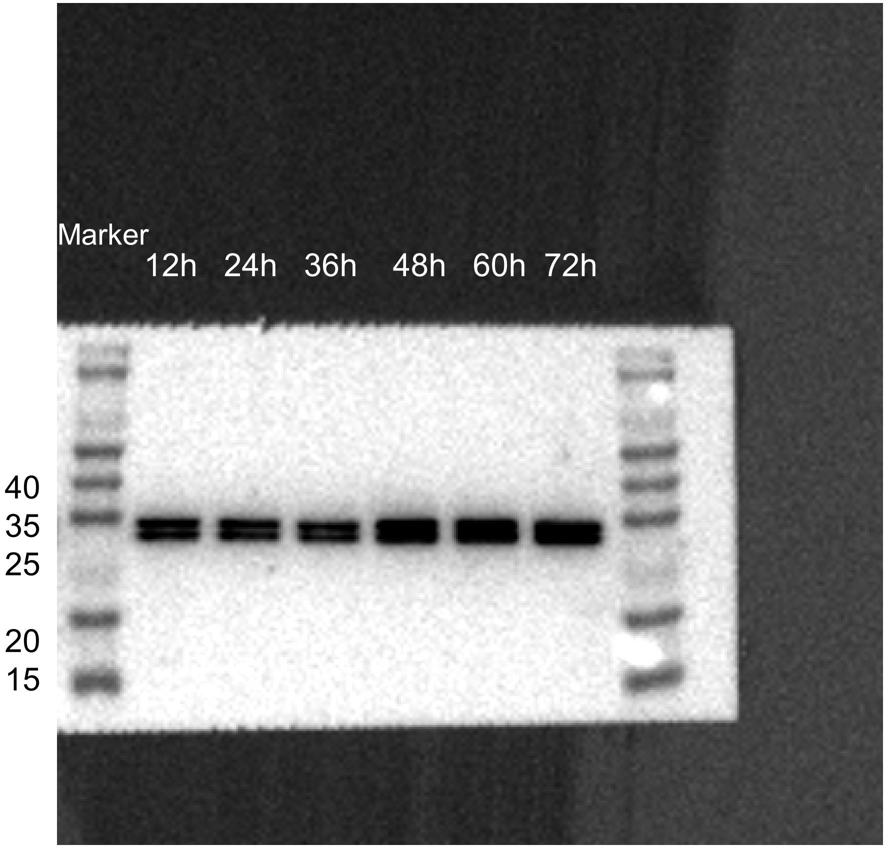

Figure 3
Procaspase-9 in empty vector–transfected cells

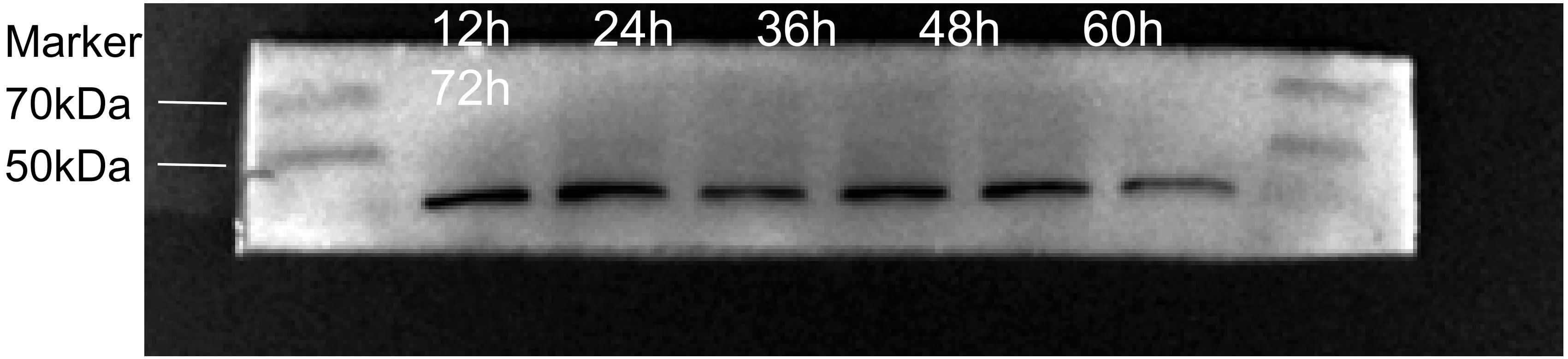

Figure3

Procaspase-8 in empty vector–transfected cells 57kDa

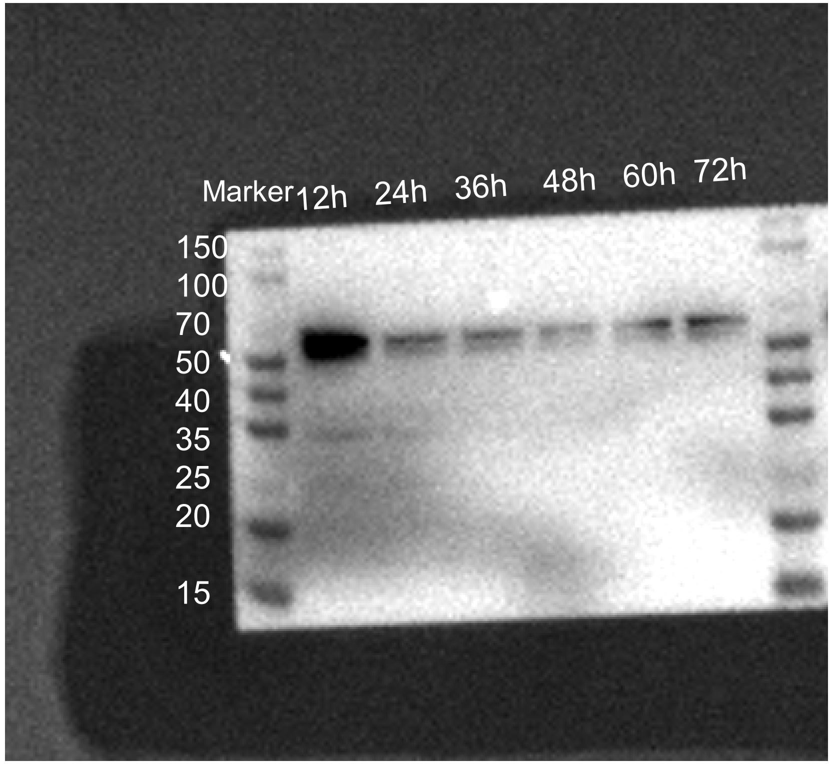

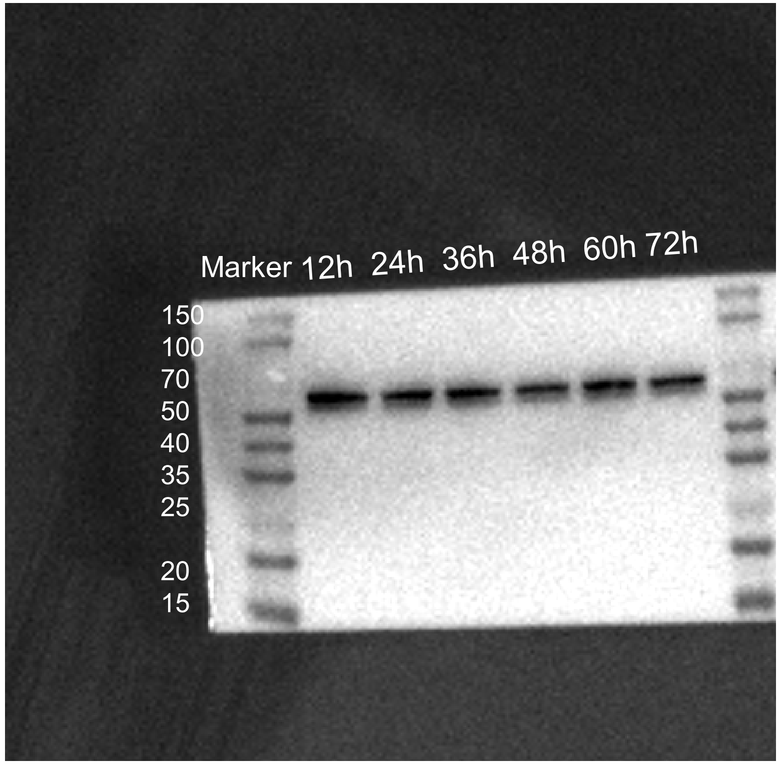

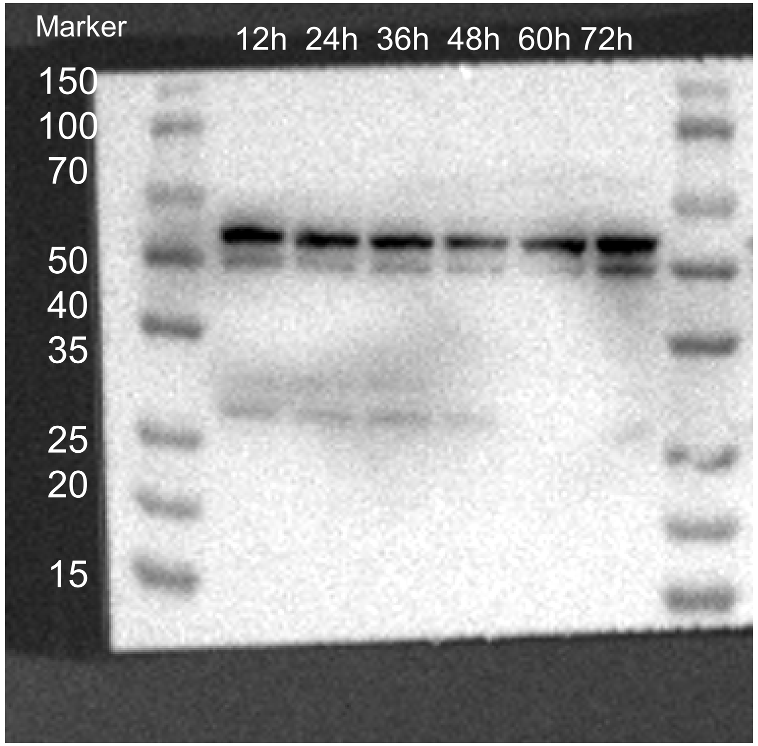

Figure 3
Procaspase-12 in empty vector–transfected cells 42kDa

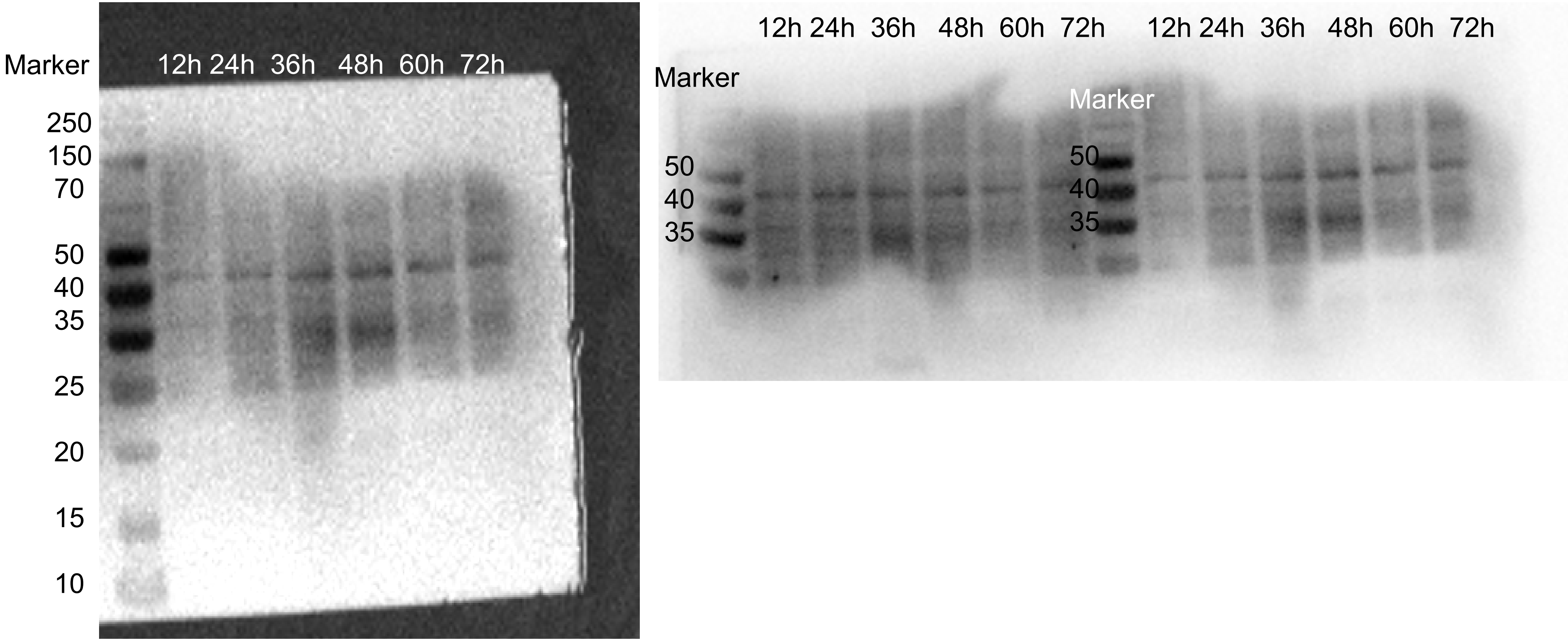

Figure 3
Cleaved PARP in empty vector–transfected cells

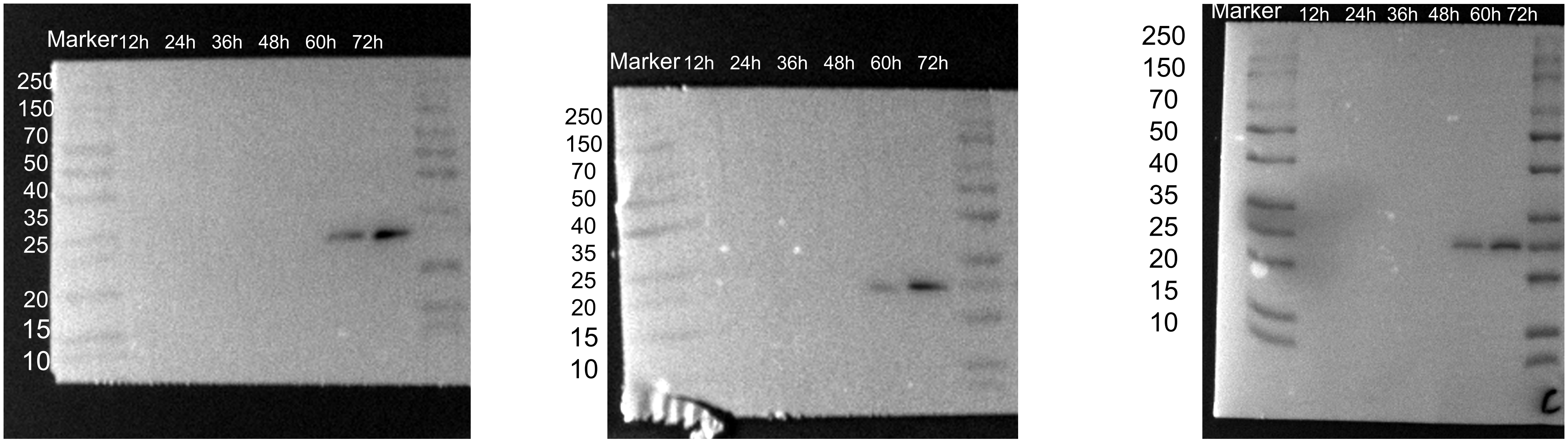

Figure 3
Procaspase-3 in positive plasmid–transfected cells 32kDa

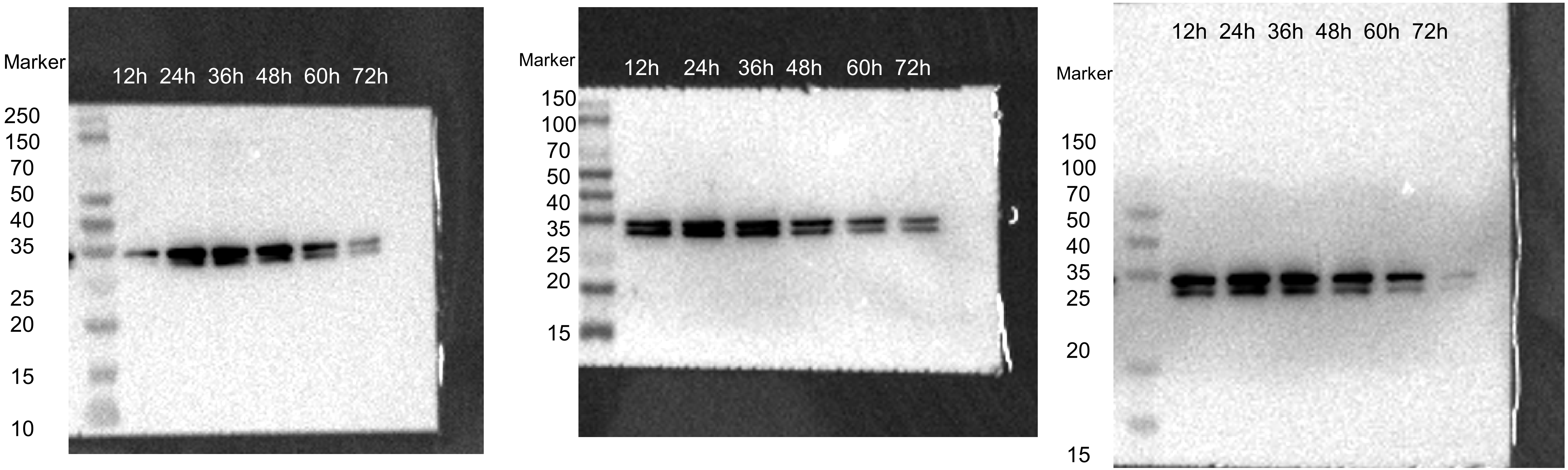

Figure 3
β-actin in empty vector–transfected cells

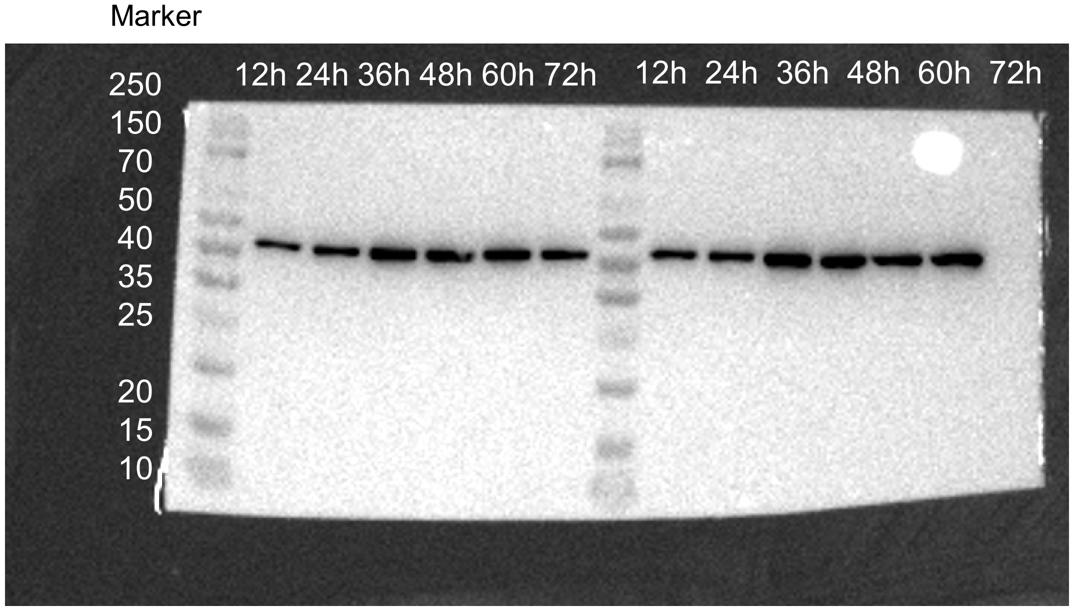

Figure 3
Cleaved caspase3 in positive plasmid–transfected cells

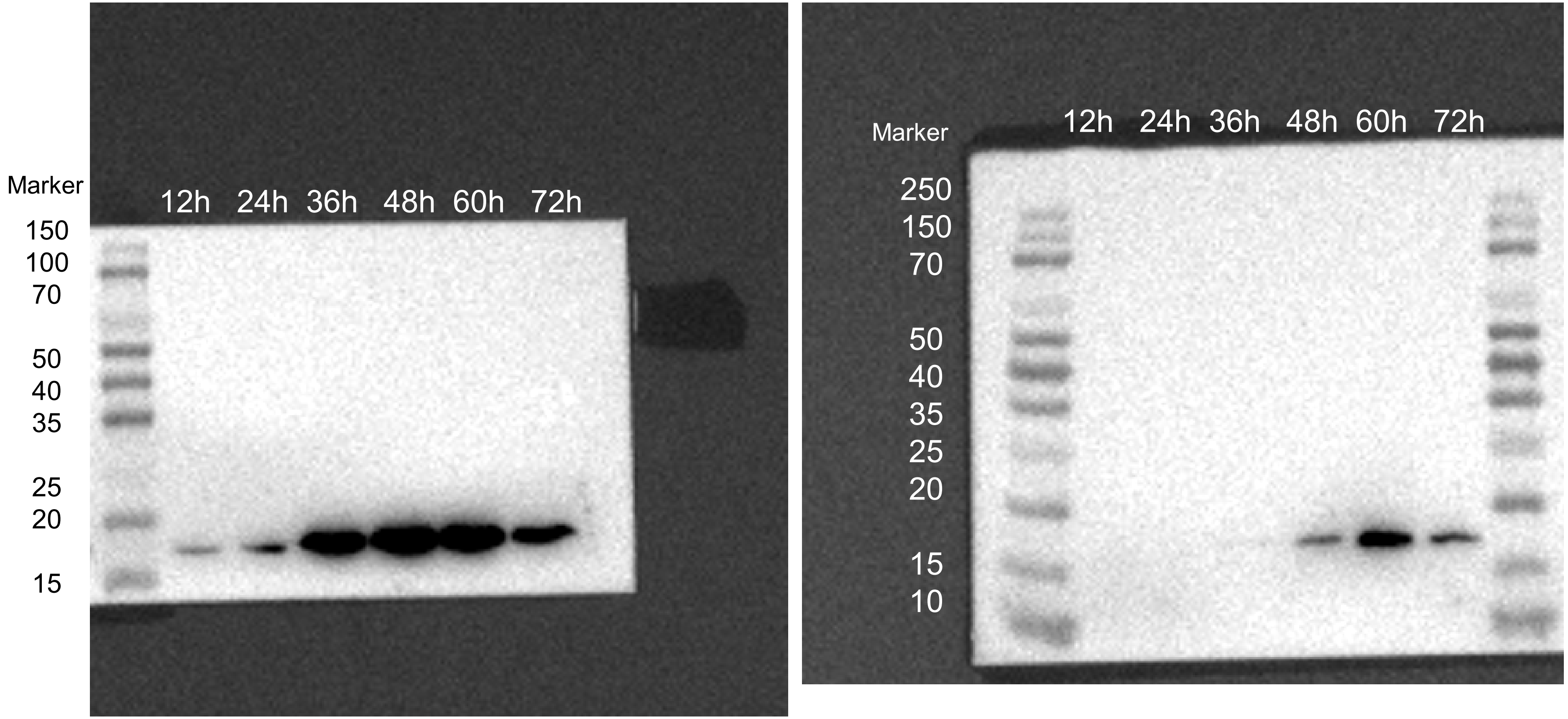

Figure 3
Procaspase-8 in positive plasmid–transfected cells 57kDa

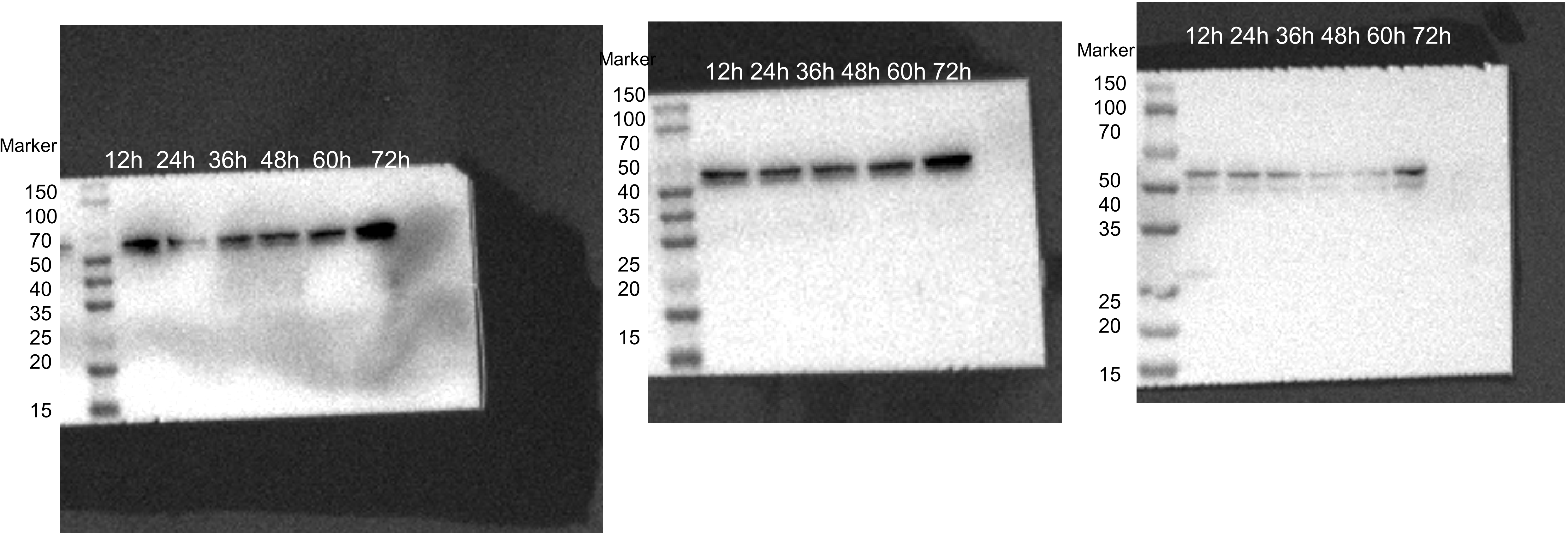

Figure 3
Cleaved caspase8 in positive plasmid–transfected cells

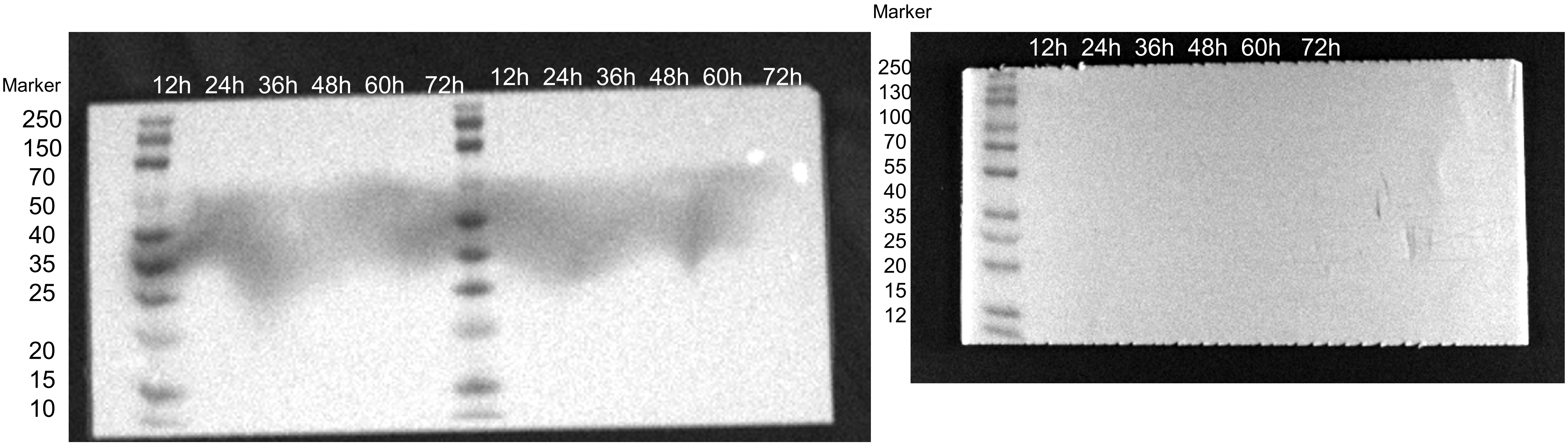

Figure 3
Procaspase-12 and Cleaved12 in positive plasmid–transfected cells42kDa

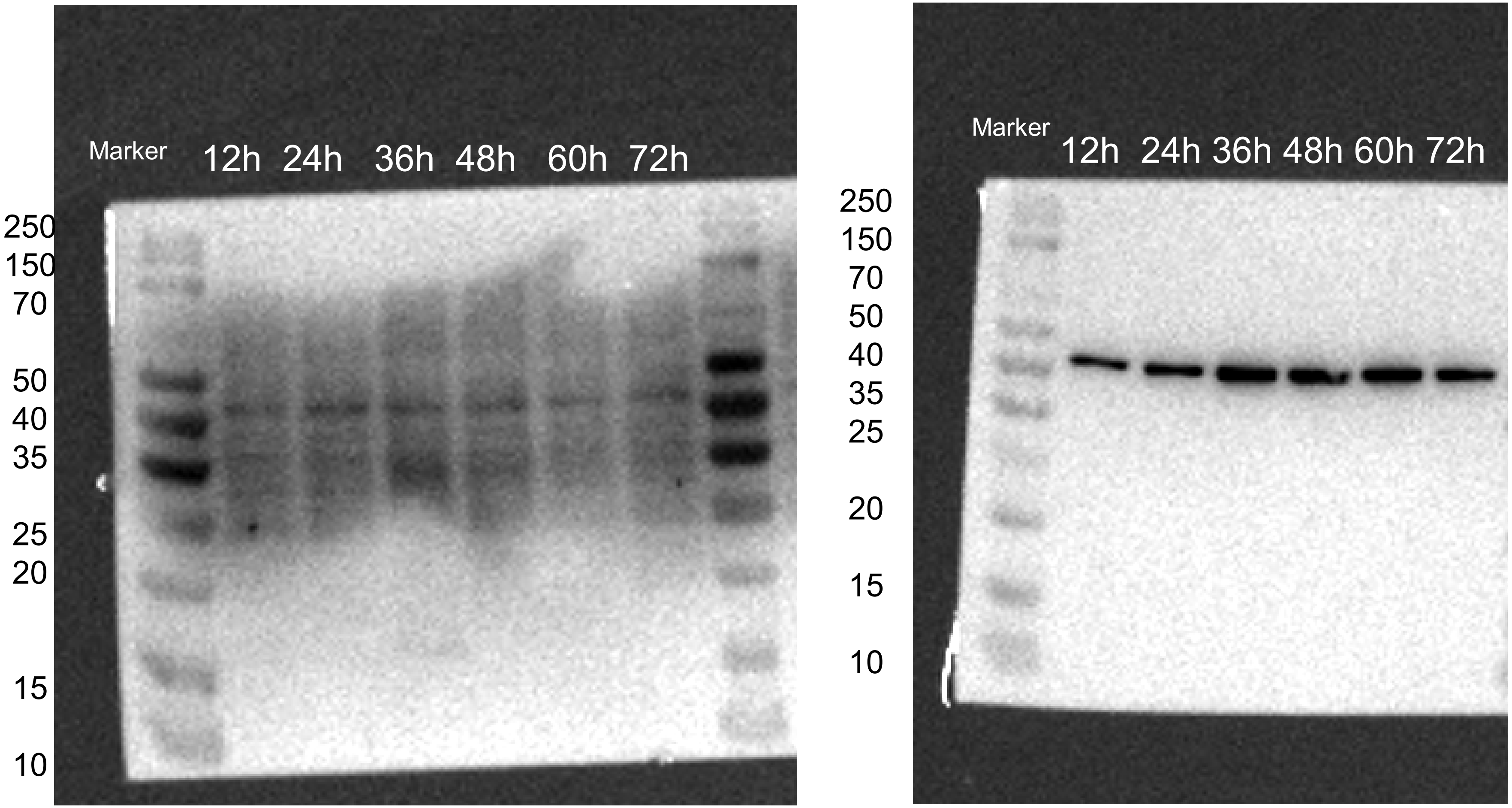

Figure 3
Procaspase-PARP in positive plasmid–transfected cells116kDa

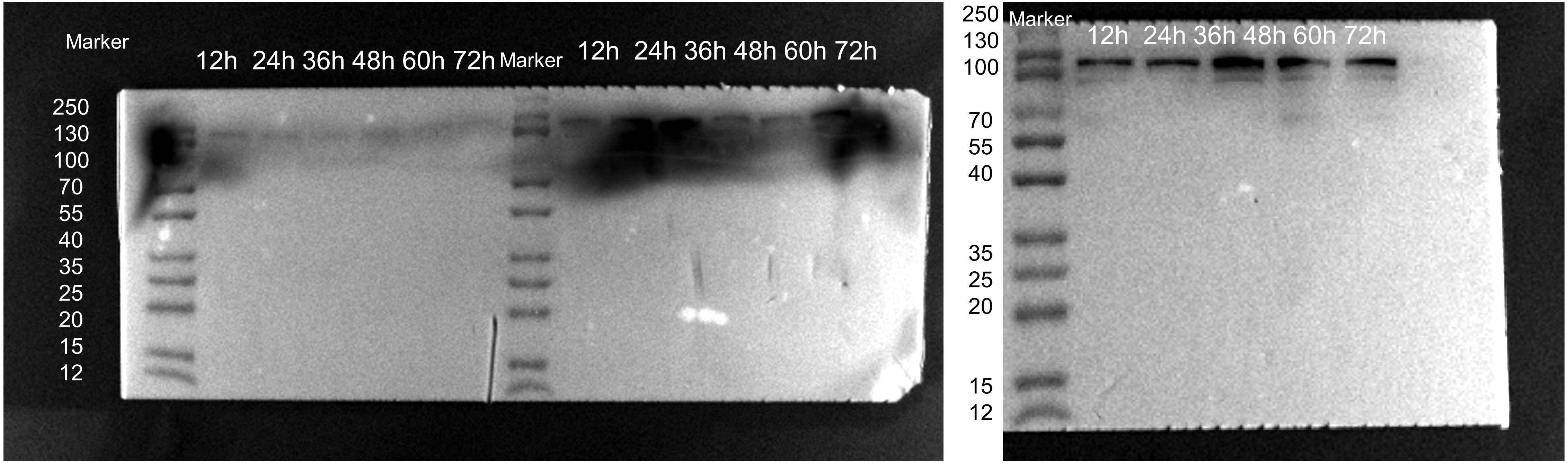

Figure 3

Cleaved PARP in positive plasmid–transfected cells

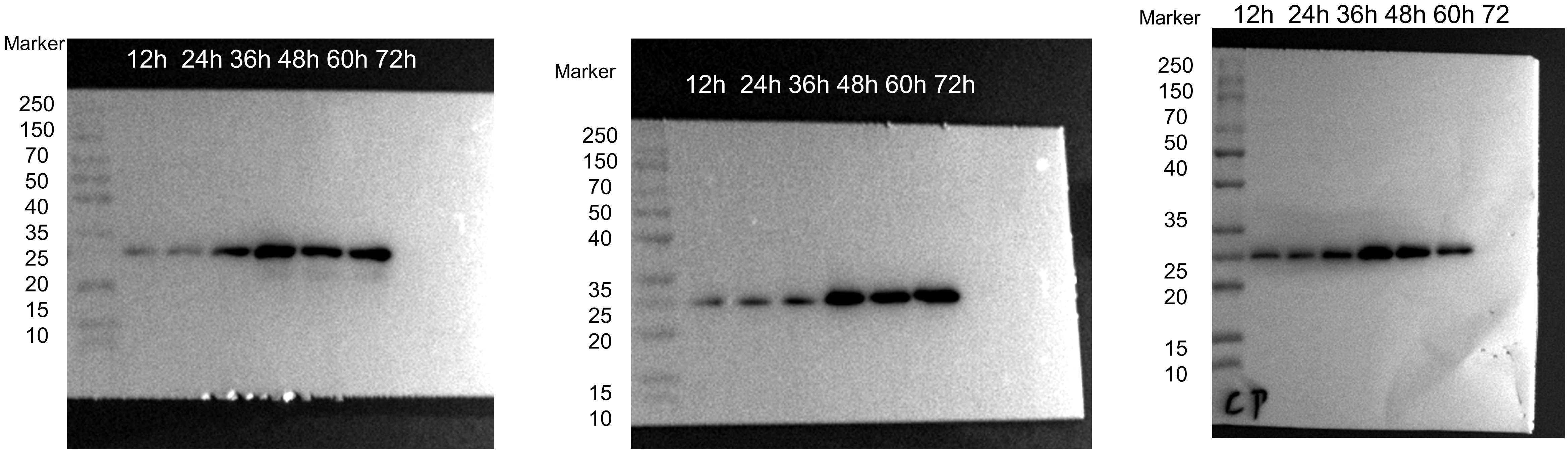

Figure 3
β-actin in positive plasmid–transfected cells

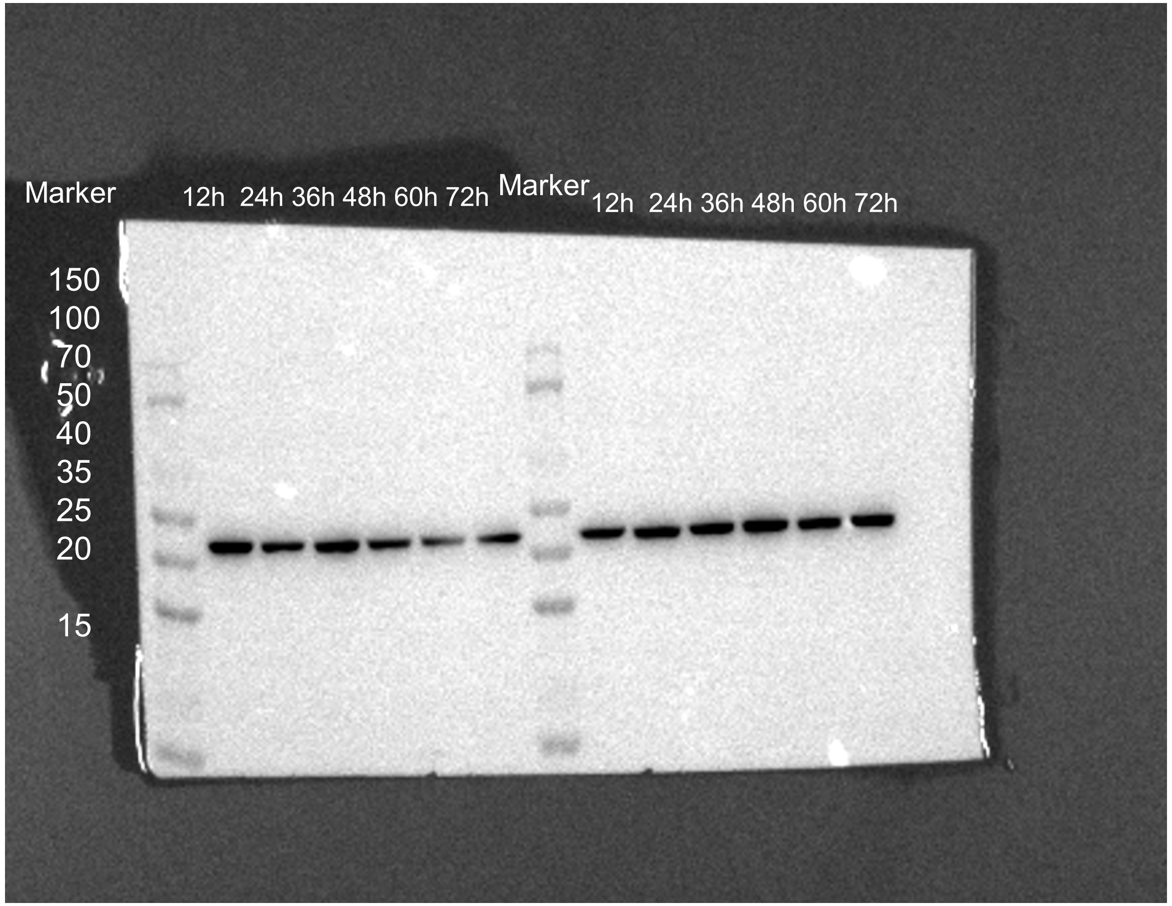

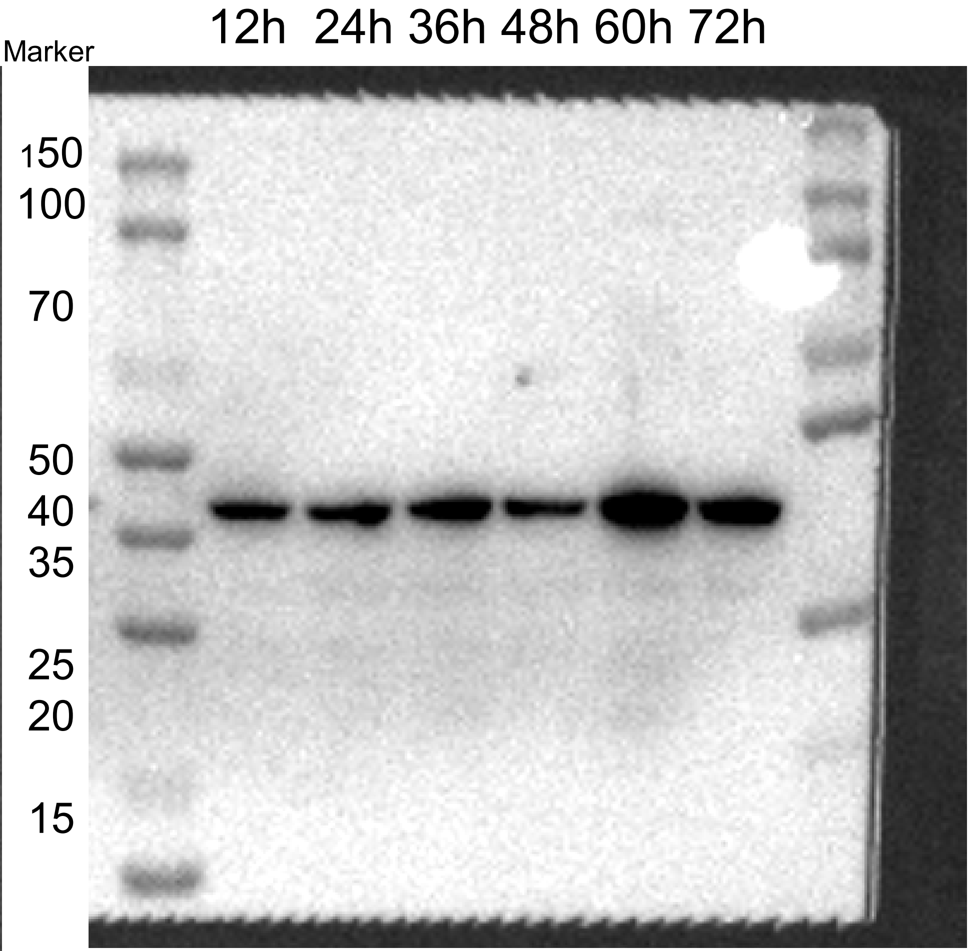

Figure 4
bcl-2 26kDa

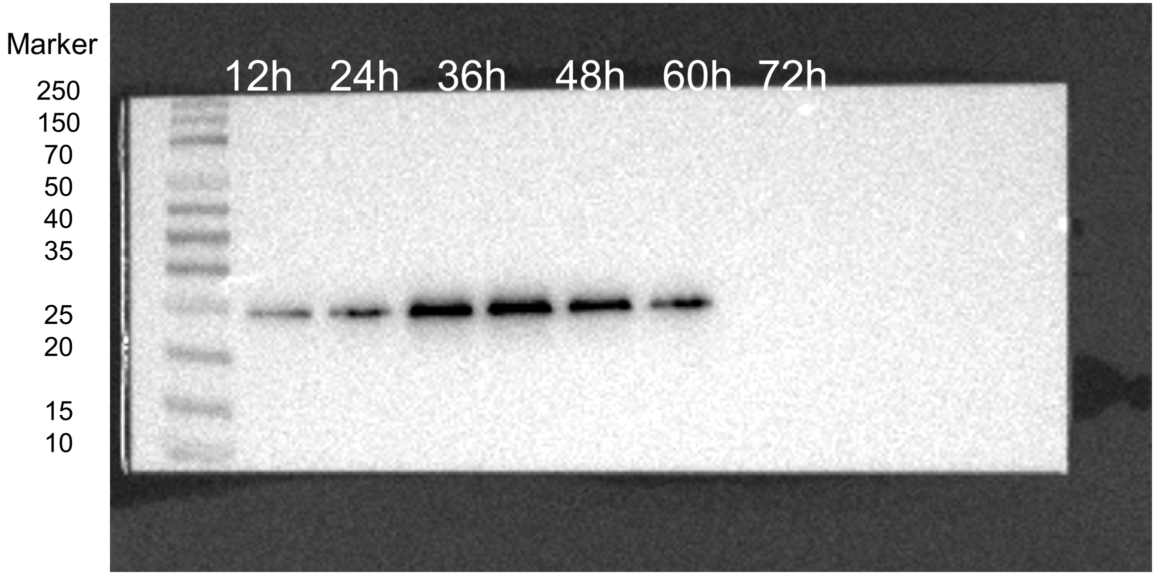

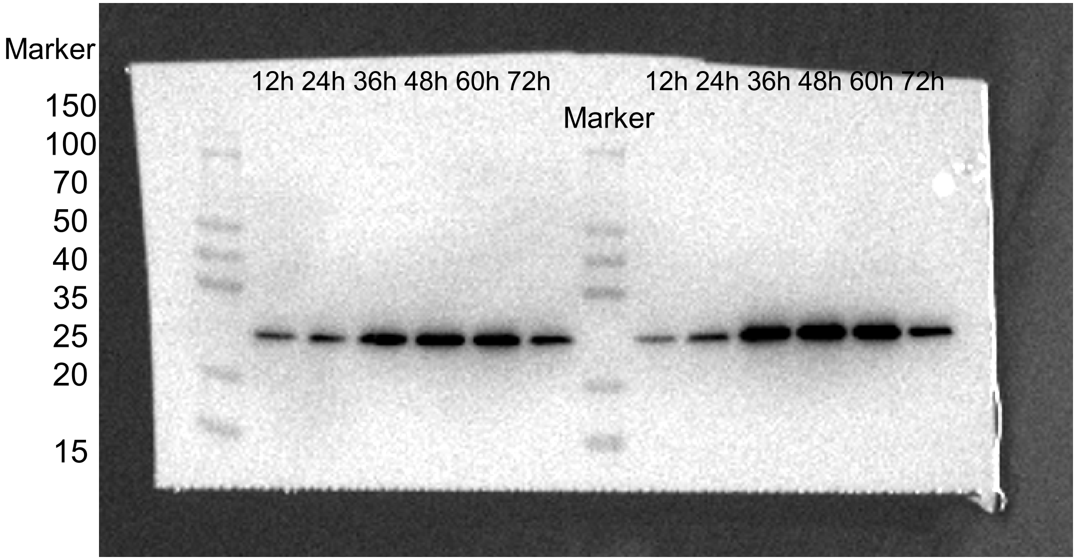

Figure 4
bax

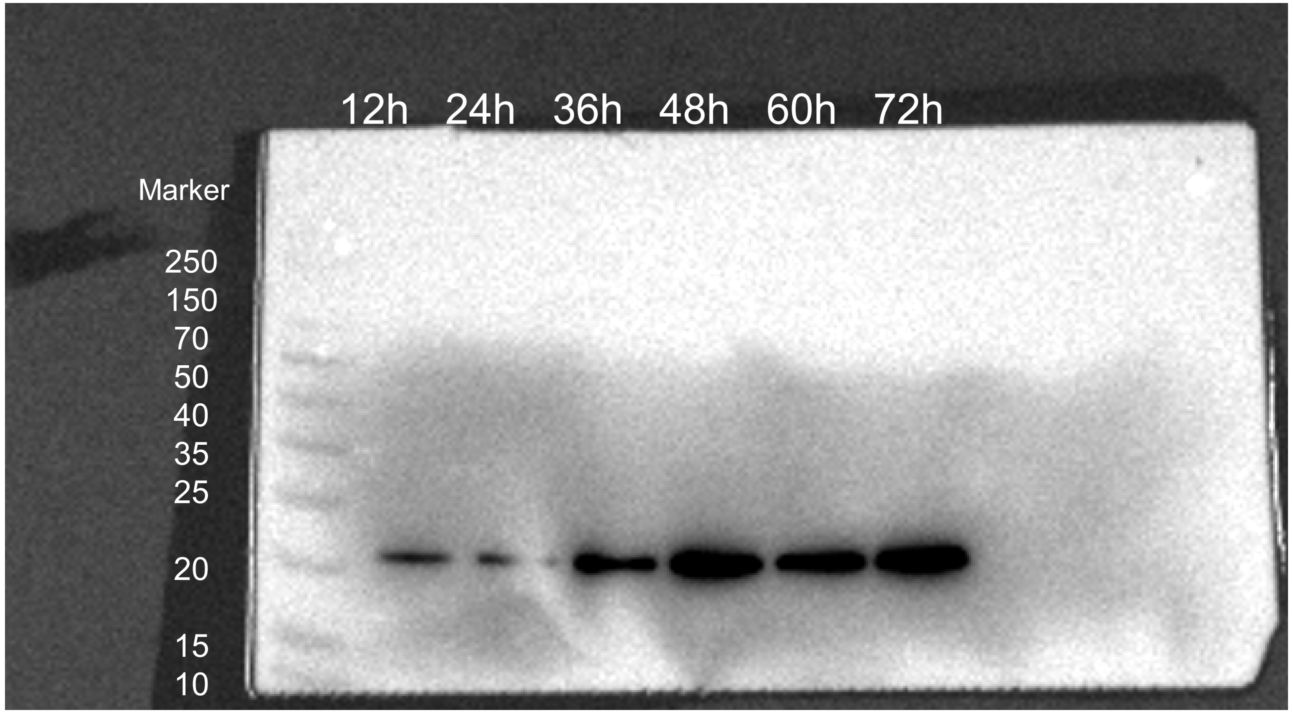
12h 24h 36h 48h 60h 72h
Marker
250
150
70
50
40
35
25
20
15
10

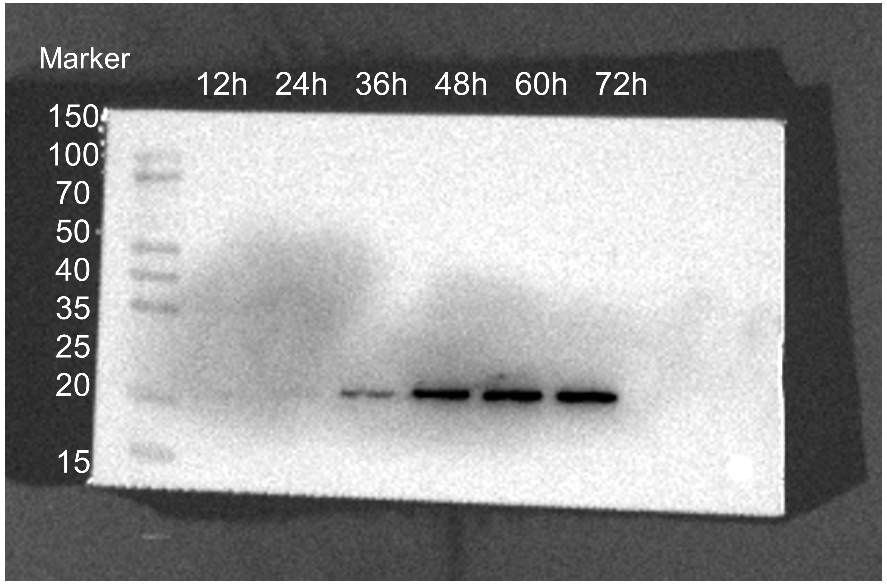
Marker
12h 24h 36h 48h 60h 72h
150
100
70
50
40
35
25
20
15

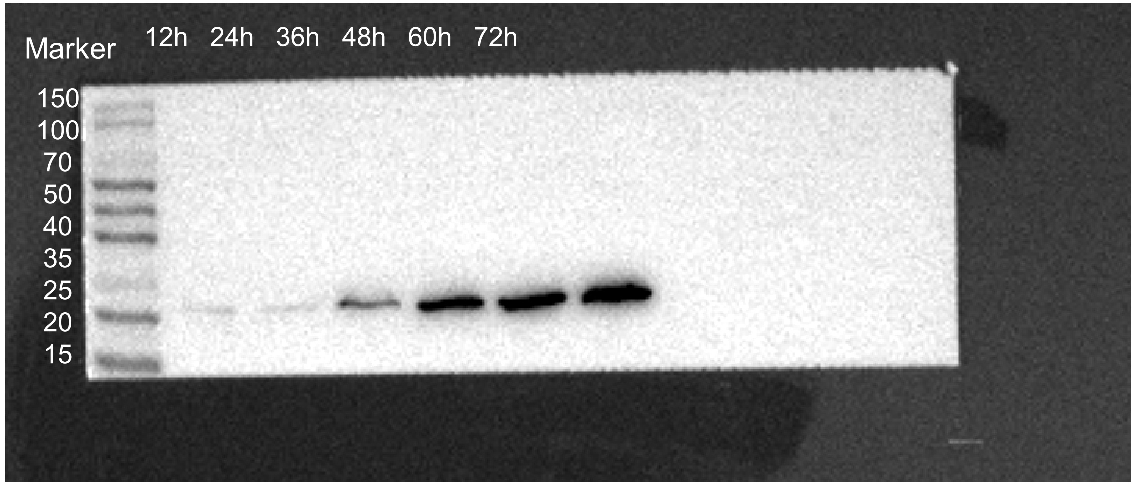
Marker
12h 24h 36h 48h 60h 72h
150
100
70
50
40
35
25
20
15

Figure 4
β-actin

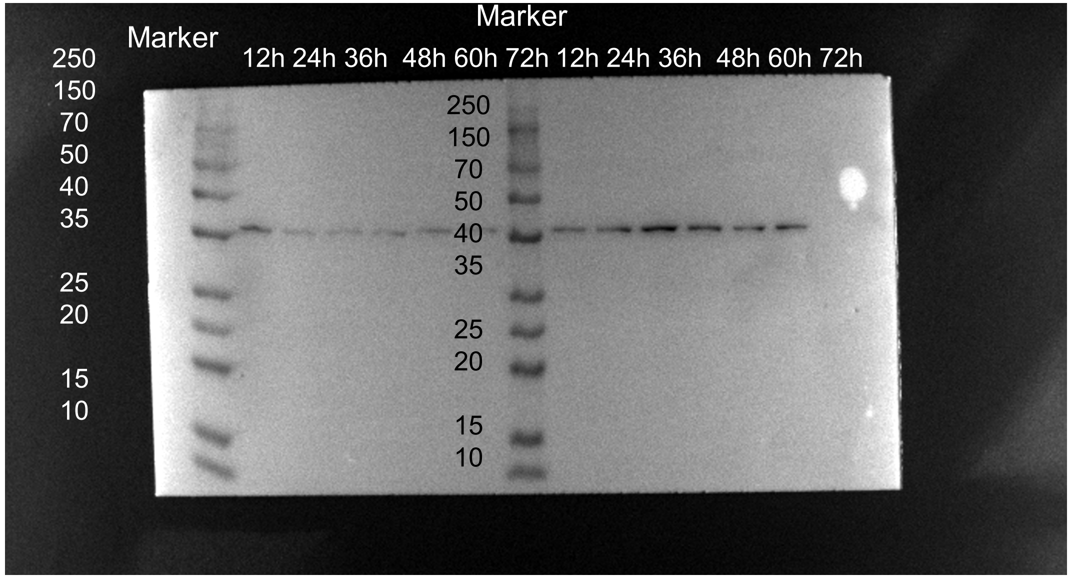

Figure 4
Cytosolic bax 25kDa

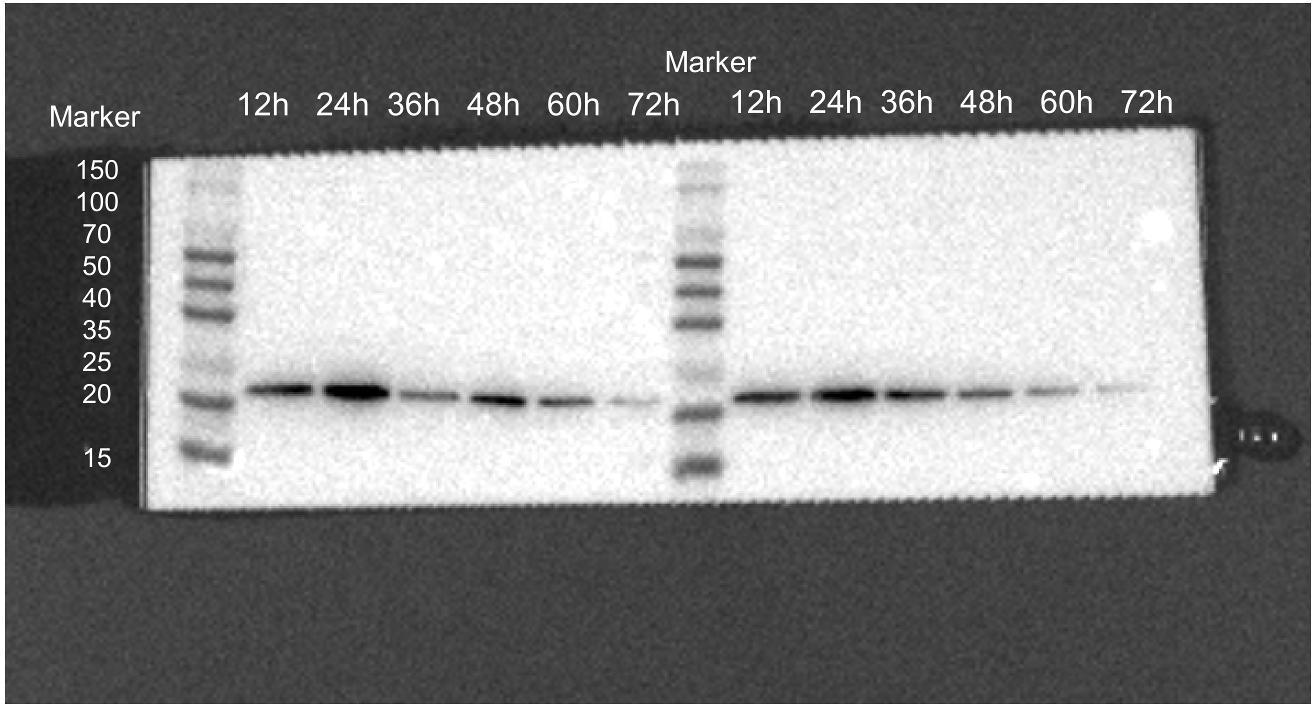

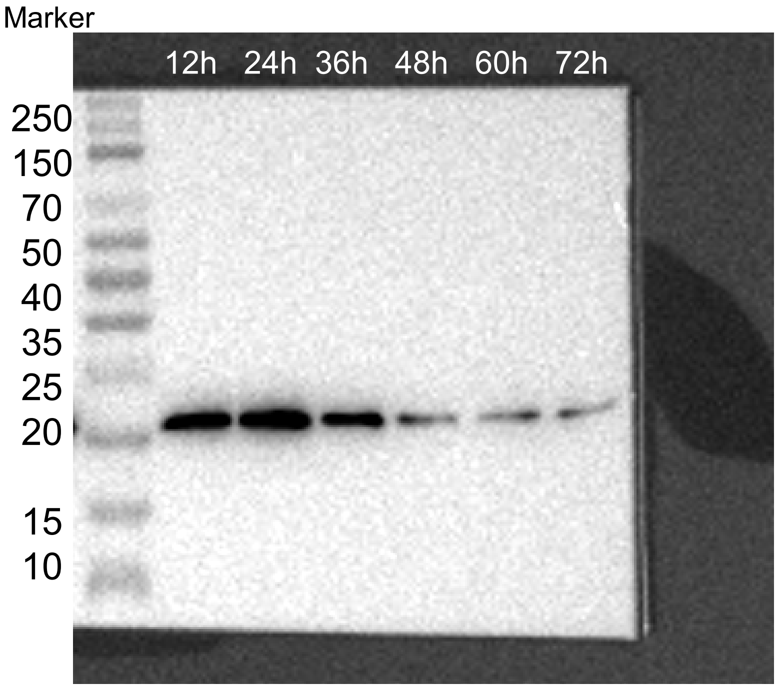

Figure 4
Cytosolic cytochrome c

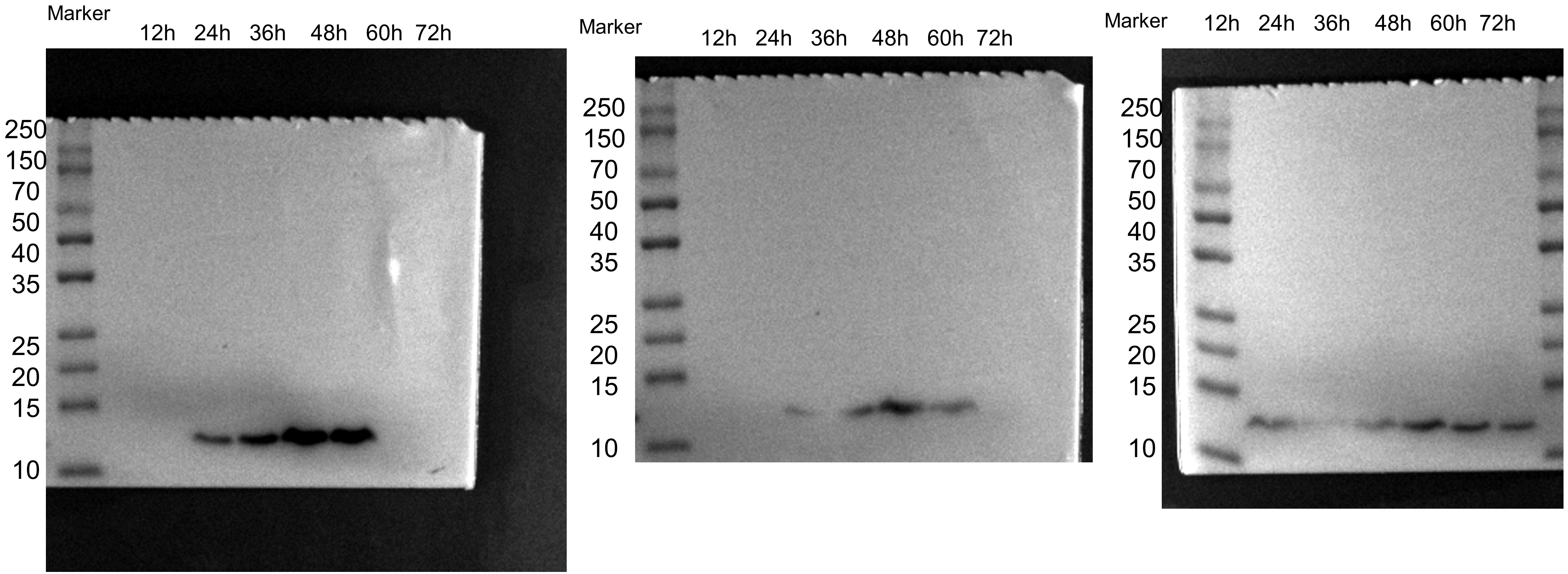

Figure 5
IP: anti-BAD; IB: anti-BAD

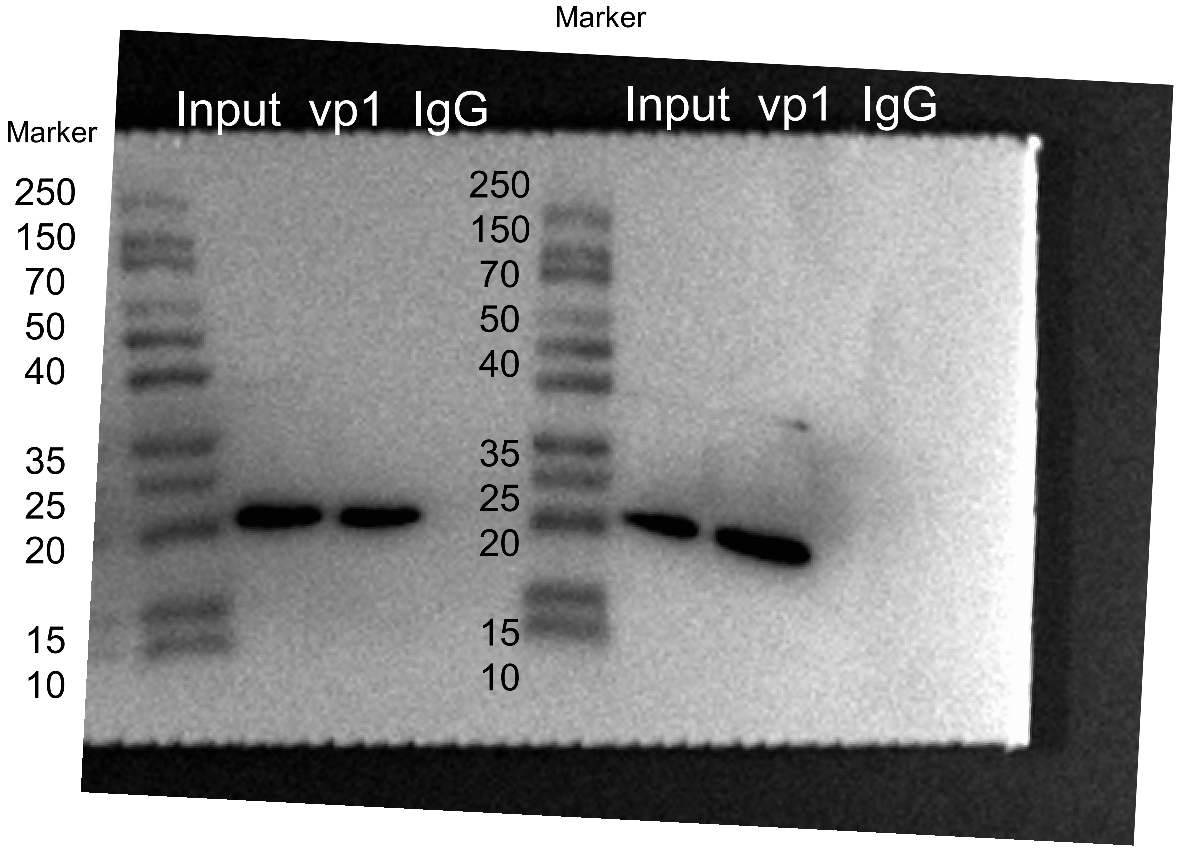

Figure 5
IP: anti-VP1; IB: anti-VP1

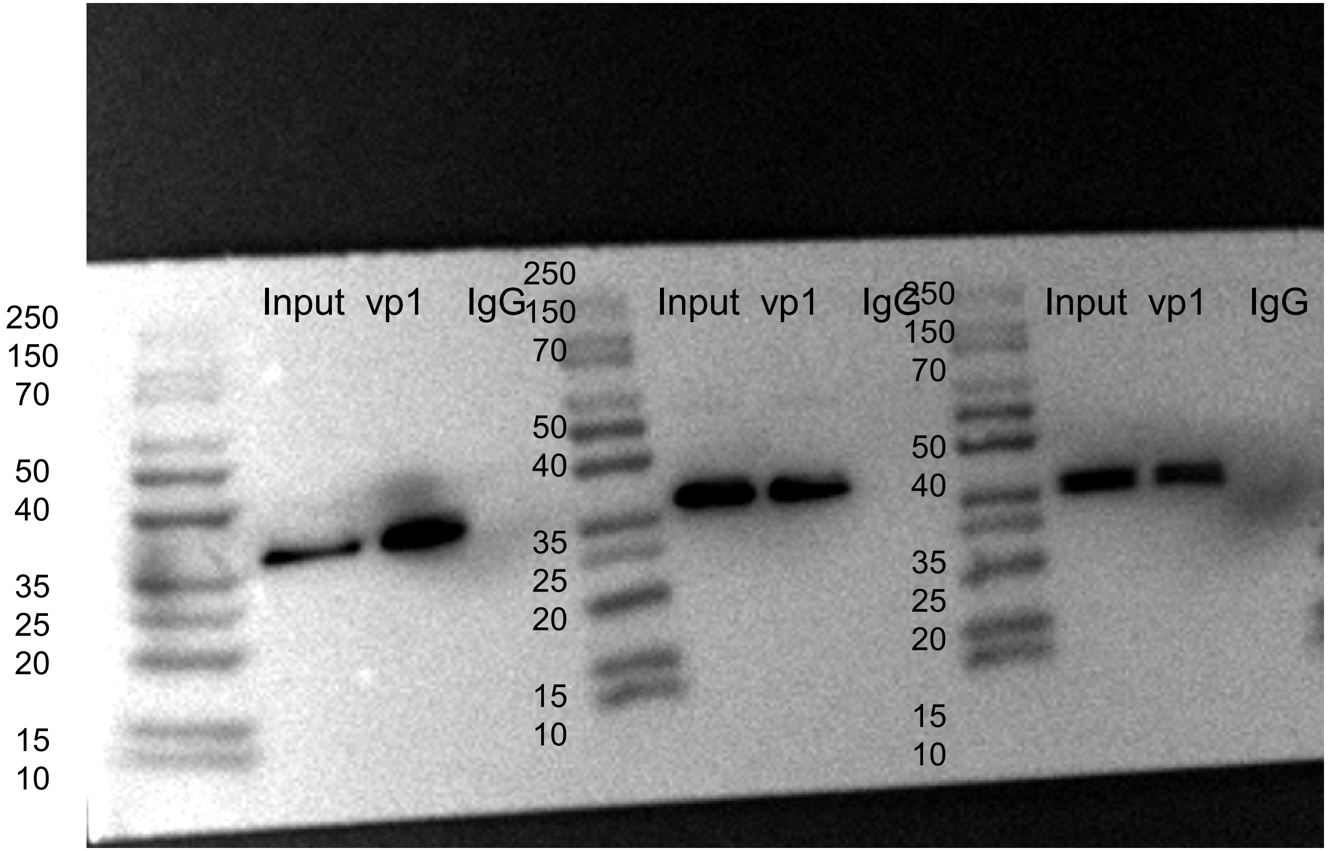

Figure 6
BAD overexpression23kDa

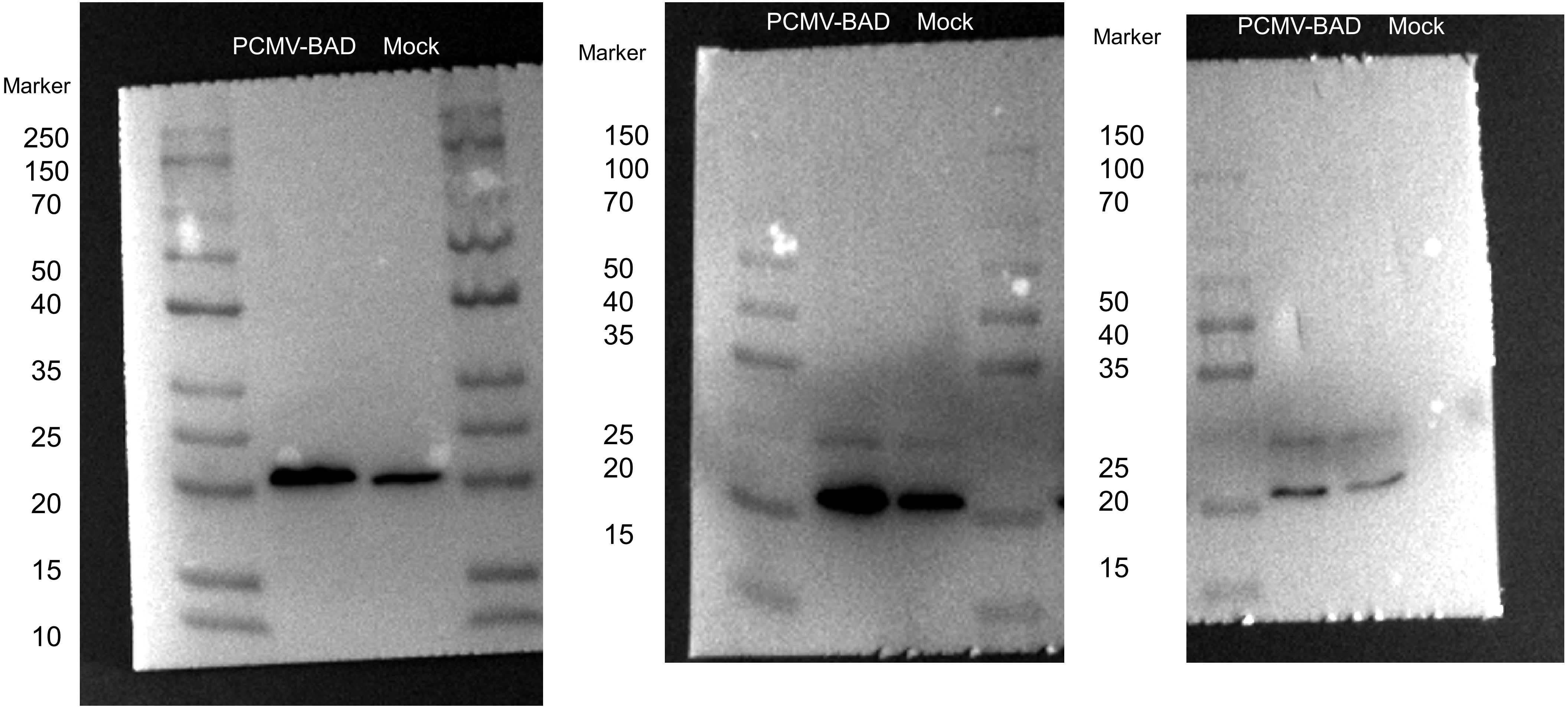

Figure 6
β-actin of BAD overexpression 42kDa

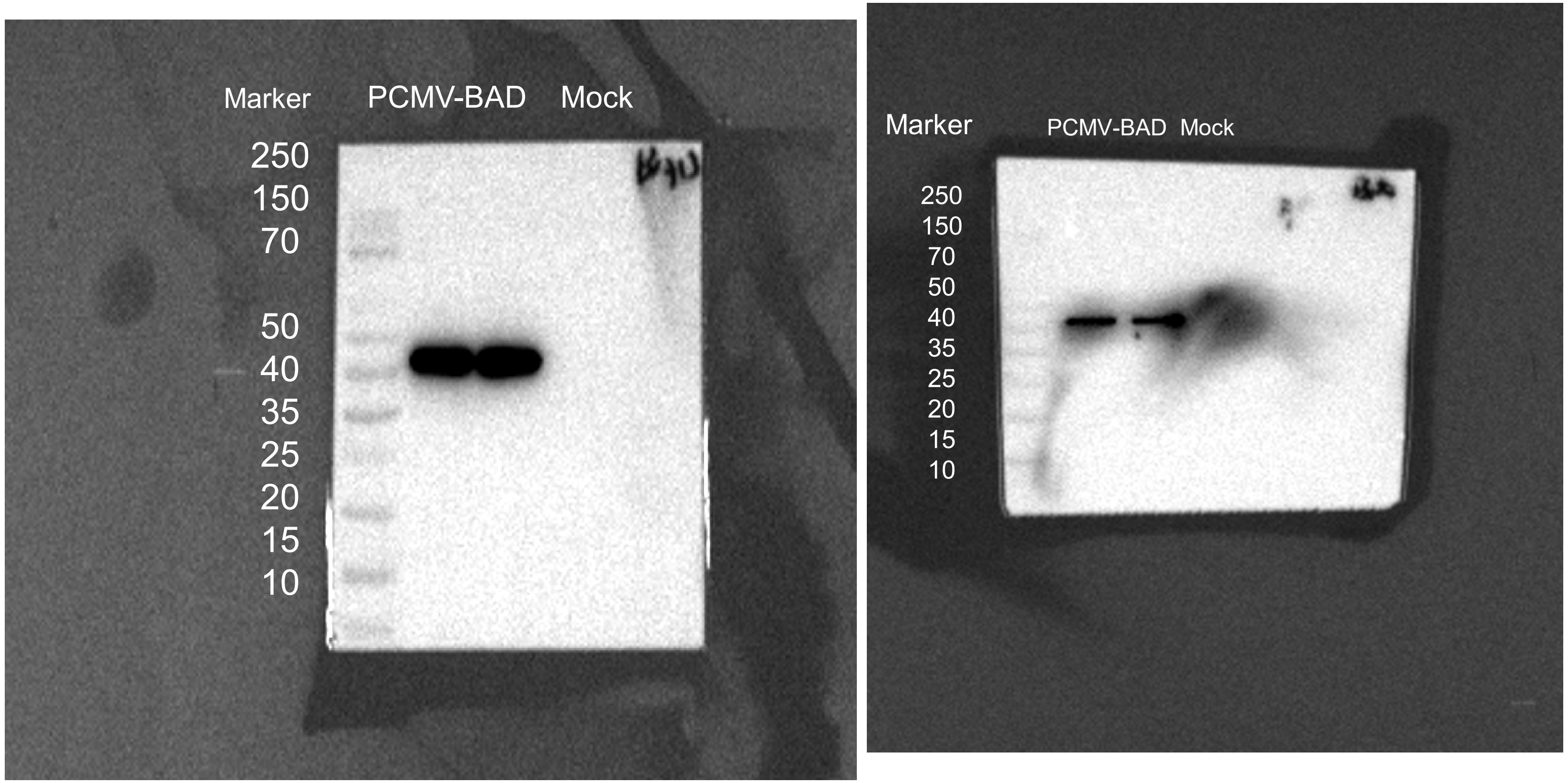

Figure 6
BAD siRNA 23kDa

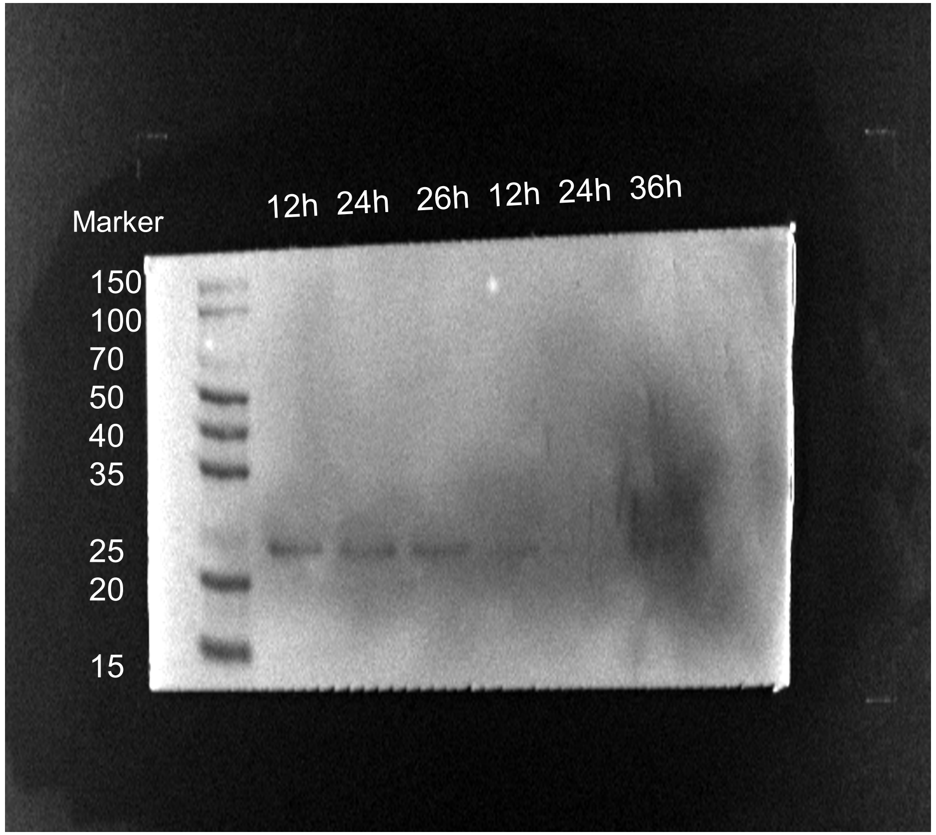

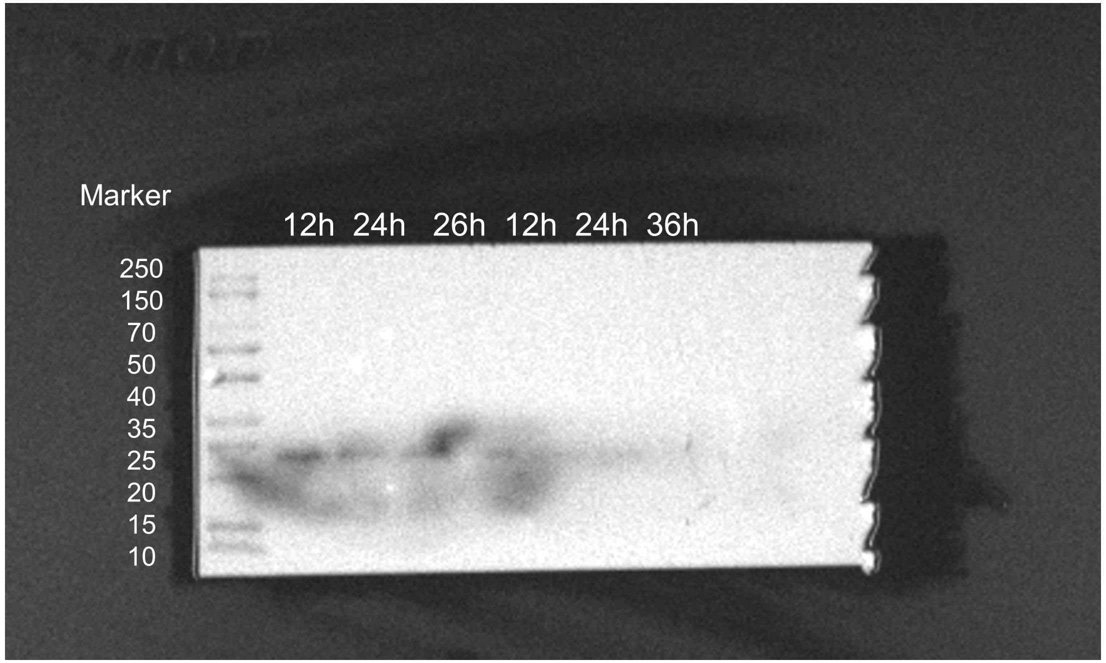

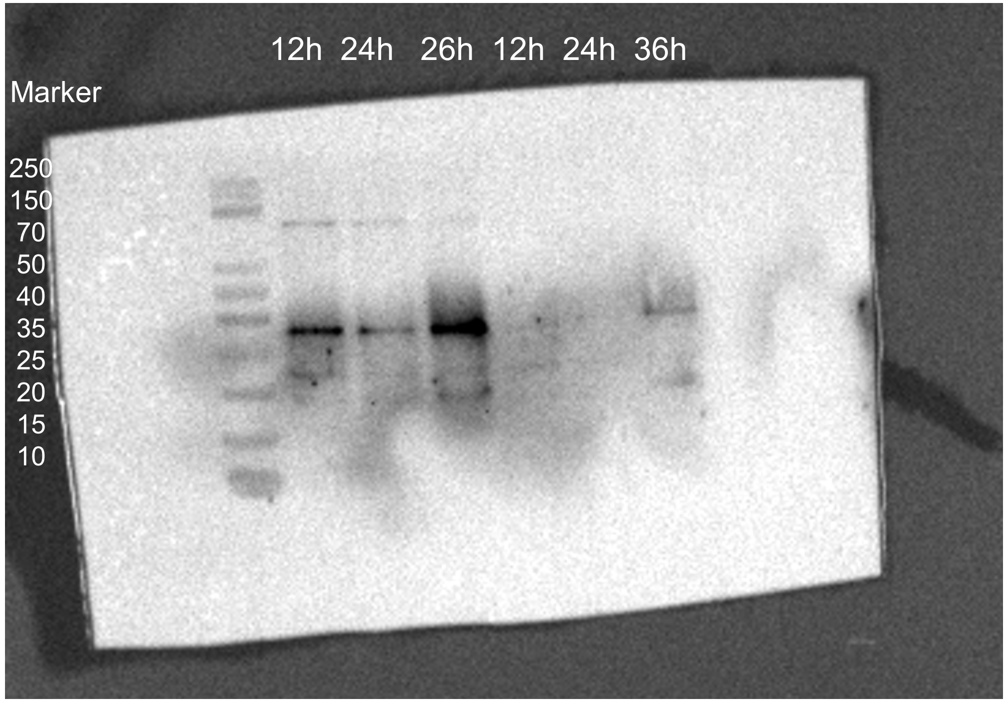

Figure 6
β-actin of BAD siRNA

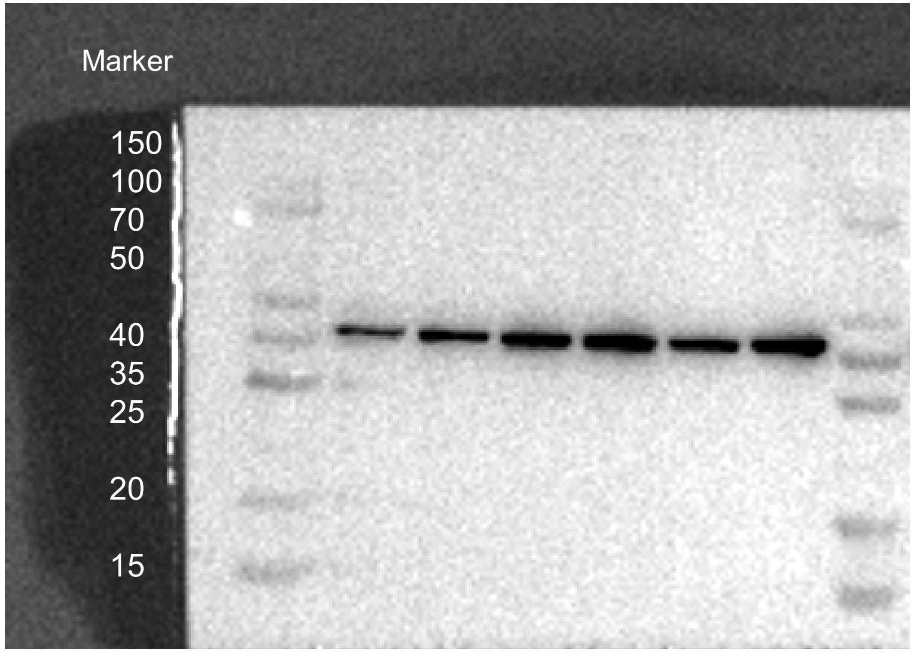

Supplement: Supplementary file 1 [file vetsci-13-00993-s001.zip › vetsci-4502506-Supplementary File/File S1/vetsci-4502506-WB-V2.pdf]
