# Supplementary material for: Caprine Kobuvirus VP1 Protein Activates the Mitochondrial Apoptotic Pathway via Interaction with BAD
Source: Vet Sci. 2026 Sep 19;13(9):993. doi: 10.3390/vetsci13090993 (PMC13612080; doi:10.3390/vetsci13090993)
Supplement: Supplementary file 1 [file vetsci-13-00993-s001.zip › vetsci-4502506-Supplementary File/vetsci-4502506-supplementary.pdf]

Table S1: Primers used in this study

Gene	Primer sequence (5'→3')	Application	Reference
**BAD**	F: TTAAGAAGGGACTTCCTCGC	qPCR	Wang, 2019
	R: CAAGTTCCTGATCCCACCAGG		
**BAX**	F: CATGGGCTGGACATTGGACT	qPCR	Wang, 2019
	R: GAGACAGGGACATCAGTCGC		
**BCL-2**	F: CTTTGAGTTTCGGTGGGGTCA	qPCR	Wang, 2019
	R: GCCGGTTCAGGTACTCAGTCA		
**AKT**	F: ACTGTCATCGAACGGCACCTT	qPCR	Wang, 2019
	R: CTCCTCCTCCTCCTGCTTCT		
**JNK**	F: GCGCAGCTTATGATGCCATT	qPCR	Wang, 2019
	R: ACTAGCTCTCTGTAGGCCCGC		
**Caspase 3**	F: ATACCAGTGGAAGGCCGACTT	qPCR	Wang, 2019
	R: ATGGCACAAAGCGACTGGATG		
**GAPDH**	F: GAGTCAACGGATTTGGTCGT	qPCR (internal control)	Wang, 2019
	R: GACAAGCTTCCCGTTCTCAG		
**CKoV 3D**	F: CGCCAAACCTGGAAGGCATCG	qPCR (viral replication)	Abi et al., 2022
	R: GGTGGAGTAAAAGTAATCTGGGTTGGA		
**CKoV 3D**	F: GGGTGAATGTCAACCGCAAAT	RT-PCR (screening)	Abi et al., 2020
	R: AGTCCCGCCGTCACTTTGG		
**VP1**	F:CCCAAGCTTGCCACCATGGCGGCTGAC GAC	RT-PCR (transfection validation)	This study
	R:CCGCTCGAGTCACTTGTCATCGTCGTCC TTGTAATCTGCACCTTGACGATGAACACA CGCAC		
